# Coupling Lipid Nanoparticle Structure and Automated Single-Particle Composition Analysis to Design Phospholipase-Responsive Nanocarriers

**DOI:** 10.1002/adma.202200839

**Published:** 2022-05-18

**Authors:** Hanna M.G. Barriga, Isaac J. Pence, Margaret N. Holme, James J. Doutch, Jelle Penders, Valeria Nele, Michael R. Thomas, Marta Carroni, Molly M. Stevens

**Affiliations:** Department of Medical Biochemistry and Biophysics Karolinska Institutet, Stockholm SE-171 77, Sweden; Department of Materials, Department of Bioengineering,and Institute of Biomedical Engineering, Imperial College London, London SW7 2AZ, UK; Department of Medical Biochemistry and Biophysics Karolinska Institutet, Stockholm SE-171 77, Sweden; ISIS Neutron and Muon Source, STFC, Rutherford Appleton Laboratory Didcot OX11 ODE, UK; Department of Materials, Department of Bioengineering,and Institute of Biomedical Engineering, Imperial College London, London SW7 2AZ, UK; Department of Materials, Department of Bioengineering,and Institute of Biomedical Engineering, Imperial College London, London SW7 2AZ, UK; Department of Materials, Department of Bioengineering,and Institute of Biomedical Engineering, Imperial College London, London SW7 2AZ, UK; Department of Biochemistry and Biophysics, Science for Life Laboratory Stockholm University, Stockholm 171 65, Sweden; Department of Medical Biochemistry and Biophysics Karolinska Institutet, Stockholm SE-171 77, Sweden

**Keywords:** enzyme-responsive systems, label-free dynamic monitoring, lipid nanoparticles, phospholipase D

## Abstract

Lipid nanoparticles (LNPs) are versatile structures with tunable physicochemical properties that are ideally suited as a platform for vaccine delivery and RNA therapeutics. A key barrier to LNP rational design is the inability to relate composition and structure to intracellular processing and function. Here Single Particle Automated Raman Trapping Analysis (SPARTA) is combined with small-angle X-ray and neutron scattering (SAXS/SANS) techniques to link LNP composition with internal structure and morphology and to monitor dynamic LNP−phospholipase D (PLD) interactions. This analysis demonstrates that PLD, a key intracellular trafficking mediator, can access the entire LNP lipid membrane to generate stable, anionic LNPs. PLD activity on vesicles with matched amounts of enzyme substrate is an order of magnitude lower, indicating that the LNP lipid membrane structure can be used to control enzyme interactions. This represents an opportunity to design enzyme-responsive LNP solutions for stimuli-responsive delivery and diseases where PLD is dysregulated.

## 1 Introduction

Lipid nanoparticles (LNPs) comprise a diverse class of versatile, bioactive structures with highly tunable physicochemical properties. Lipid composition can be used to tune the morphology and function of LNPs to produce particles with a broad range of morphologies including ordered and disordered lipid structures.^[1,2]^ Demonstrating unparalleled performance as delivery vehicles, three LNP formulations are currently approved for human use: two received emergency use authorization for mRNA delivery in COVID-19 vaccines, while the third is an siRNAbased amyloidosis treatment.^[3,4]^ As only a small fraction of administered LNPs elicit a functional response, there is an exceptional need to improve the understanding of LNP biodistribution, uptake, endosomal escape, and intracellular processing to expand their applications within biomedicine.^[5–8]^ The most highly reported LNP compositions are thought to have amorphous lipid cores and use remarkably similar lipid compositions which include ionisable lipid, cholesterol, phospholipid, and a PEGylated lipid.^[1]^ To expand the scope of LNP formulations and the mechanisms underpinning their function, further investigation of novel formulations and structures is needed.

Optimization of LNP formulations has focused predominantly on composition screens that provide limited structural information on the resulting LNPs.^[9–11]^ Small compositional variations can lead to significant differences in LNP structure, function, and biodistribution.^[7,11–13]^ Multiscale characterization of novel LNP formulations with structured cores is essential to connect LNP composition and structure to functional performance and the key focus of this publication.

**Figure 1:**
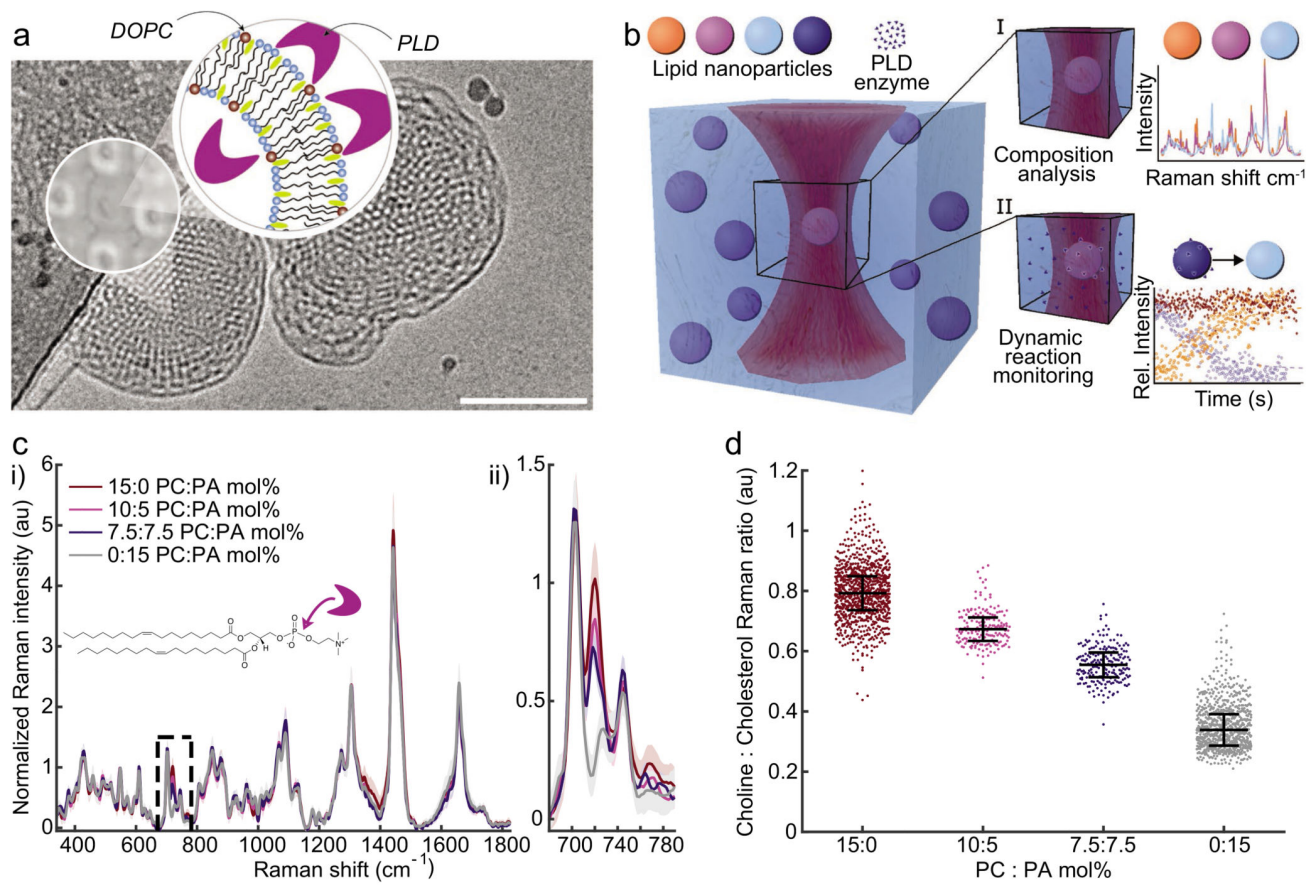
Techniques for single-particle analysis of phospholipase D conversion of DOPC (PC) to DOPA (PA) in LNPs. a) Schematic of PLD interaction with LNPs including a representative Cryo-TEM of PC-containing LNPs (MO:CHOL:DOPC 55:30:15 mol%) formulated with 2.5 wt% F127. Scale bar: 100 nm. The inset schematic depicts the lipid membrane (bicontinuous cubic, Im3m) with PLD enzyme and the approximate ratios of lipid components where DOPC = brown, monoolein = blue, and cholesterol = yellow. b) SPARTA schematic for single-particle evaluation for LNP composition analysis and dynamic processes monitoring. c) (i) Example Raman spectra (mean ± standard deviation, *n* ≥ 175) obtained from individual LNPs comprising compositions MO:CHOL:DOPC:DOPA 55:30:15-*X:X* mol% where *X* = 0, 5, 7.5, 15 mol% (ii) the choline peak at 718 cm^−1^ demonstrating the ability to detect composition differences between single LNPs at 25 °C. d) Quantification of the choline signal of individual particles for distinct LNP formulations normalized to cholesterol signal (718/703 cm^−1^) (median ± IQR, *n* ≥ 285). Inset structure rendering in (a): Adapted under the terms of the CC-BY Creative Commons Attribution 4.0 International license (https://creativecommons.org/licenses/by/4.0).^[33]^ Copyright 2015, The Authors, published by Springer Nature.

Phospholipases are key mediators in intracellular trafficking and can alter the composition and structural integrity of lipid delivery vectors.^[14]^ Phospholipase D (PLD) enzymes cleave choline headgroups from phospholipids, forming membrane-bound anionic phosphatidic acid and releasing choline which can act as a secondary messenger (Figure 1a). Dysregulation of PLD activity has been observed in numerous disease pathways including neurodegeneration, vascular disease, and cancer (breast, colon, and brain).^[15,16]^ PLD activity and the resulting downstream signaling is also highly dependent on isoform, protein interaction, calcium concentration, and lipid composition.^[17–23]^ Whilst composition screens have elucidated the importance of LNP phospholipid components,^[9]^ significant knowledge gaps persist regarding LNP interactions with phospholipases, the resulting effects on the LNP lipid composition and structure, and the potential to harness these changes for stimuli-responsive delivery or sensing.

Lipid assemblies, including LNPs, can be characterized using small-angle X-ray scattering (SAXS) and small-angle neutron (SANS) to probe the bulk structure of formulations and quantify the effects of lipid composition,^[24,25]^ hydration,^[26]^ pressure/temperature,^[27]^ the location of LNP components^[28,29]^ and payload loading.^[30]^ SAXS/SANS experiments enable exploitation of the high SAXS resolution for phase behavior determination via electron rich interactions and the SANS high contrast generated from exploitation of scattering length density differences in hydrogen and deuterium.^[31]^ The combination of the two techniques offers a powerful tool to probe the structure of lipid systems. However, they cannot provide specific information regarding sample composition or monitor rapid reaction dynamics.

Single Particle Automated Raman Trapping Analysis (SPARTA), previously demonstrated on a variety of nano-particles,^[32]^ provides rapid, label-free composition-specific measurements of individual LNPs enabling the interrogation of population dynamics over time. As depicted in Figure 1b, SPARTA combines optical trapping and Raman spectroscopy to capture and hold a target LNP while simultaneously acquiring a high-resolution spectrum comprising the LNP’s molecular vibrations. SPARTA provides comparative quantification of subtle changes in LNP compositions, including those mediated by PLD, (Figure 1c,d).

Here, we implement SPARTA, small-angle scattering (SAXS/SANS) and fluorescence assays, to investigate the composition and structural response of DOPC-containing LNPs to PLD activity in comparison to PLD activity on vesicles. We assess specific alterations in structure and composition based upon formulation and environment (temperature), and explore the morphology-dependent activity of PLD independent of LNP compositions. Our approach underscores that by interrogating bioactive LNPs, both as single particles and in bulk, stimuli-responsiveness (i.e., temperature, enzyme interactions) can be precisely measured and controlled, facilitating improved rational design for myriad biomedical applications.

## 2 Results and Discussion

### 2.1 PLD Can Access the Entire LNP Membrane during Active Substrate Conversion of Single Particles

**Figure 2:**
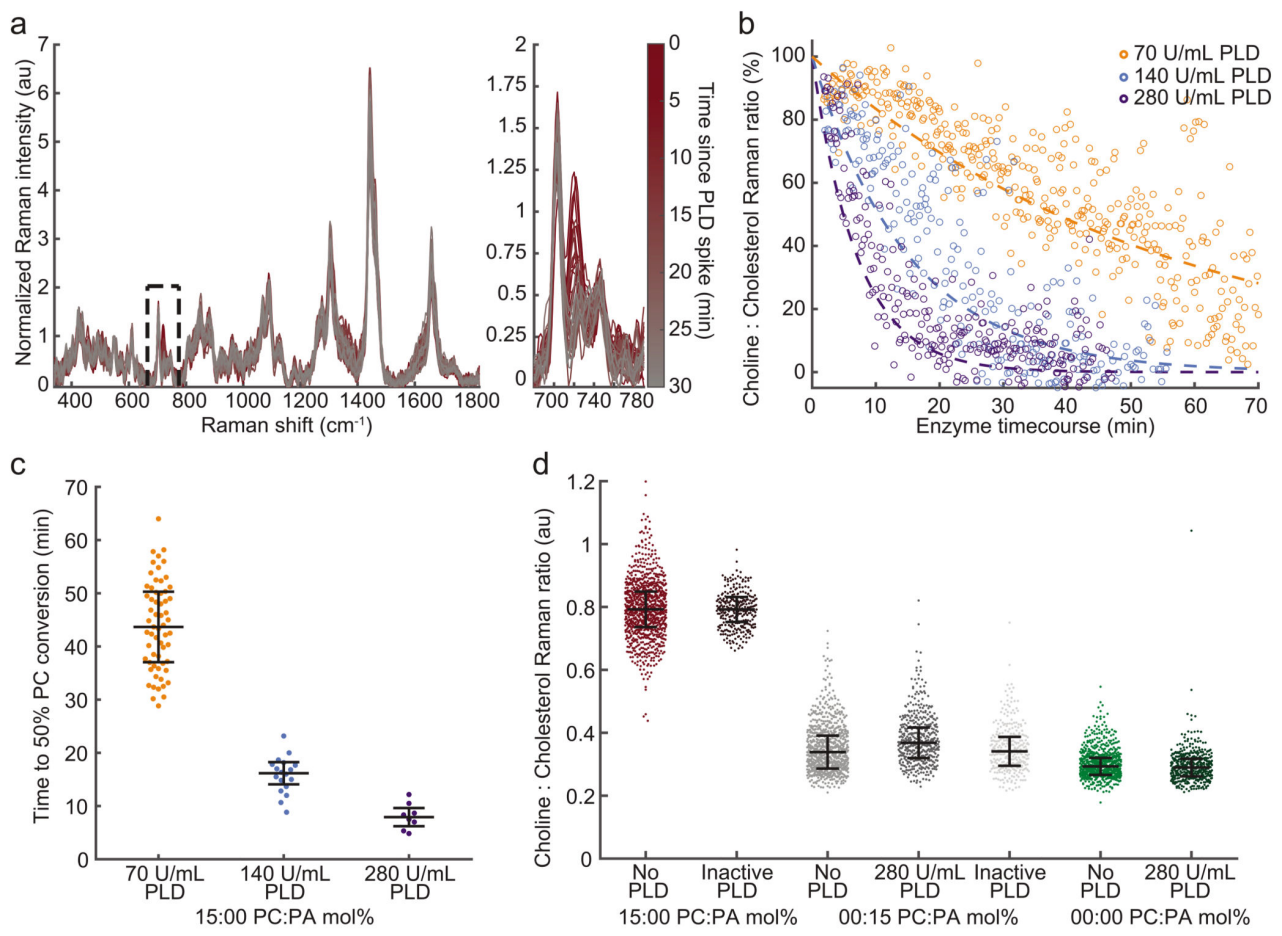
Detection of phospholipase D conversion of single LNPs using SPARTA to measure composition and dynamics as a function of enzyme concentration at 25 °C. a) Spectra recorded from a single experiment of individually trapped MO:CHOL:DOPC 55:30:15 mol% LNPs after addition of 280 U mL^−1^ PLD over a 30 min time-course with choline:cholesterol region magnified (line color representative of time-course experiment). b) Normalized change in choline:cholesterol signal for MO:CHOL:DOPC 55:30:15 mol% LNPs trapped after addition of 280, 140, and 70 U mL^−1^ PLD. Each data point represents a single measurement and a new trapping event while the dashed lines represent first order kinetics fit of PLD activity. c) Time to 50 mol% conversion of PC to PA by PLD activity for each enzyme concentration (median ± IQR, *n* = 61, 18, and 8, respectively). Each data point corresponds to the experimental time since the addition of PLD for a single particle with choline:cholesterol between 45−55% based on control formulations with 15:0 mol% and 0:15 mol% PC:PA (Figure 1c,d). d) Quantification of the cholesterol normalized choline signal (718/703 cm^−1^) (median ± IQR, *n* ≥ 285) of individual particles for control LNP formulations with no PLD and with the addition of native and deactivated PLD (mol% Ch =30). Expected heterogeneity of individual LNPs can be observed while PLD activity is only present for experiments with both active enzyme and LNP formulations containing the PC substrate.

LNPs with an internal bicontinuous cubic phase morphology comprise a network of water channels separated by a lipid bilayer and are capable of storage and release.^[34–36]^ However, access to the water channels in bicontinuous cubic phases remains unclear.^[33,37,38]^ Based upon the specific signatures of the constituent nonpolar lipids, we show that for this LNP formulation, both water channels are accessible to PLD enzyme in solution. Signals obtained from individually trapped LNPs (MO:CHOL:DOPC 55:30:15 mol%) measured sequentially over 30 min following the addition of PLD show a characteristic loss of the choline peak signal (718 cm^−1^) via hydrolytic action of the enzyme (Figure 2a). Direct comparison and statistical analysis of the presented time-course and the control formulations (Figure 1d) with known mol% DOPC indicates these LNPs are completely converted from DOPC to DOPA over this time-course, exhibiting cleavage of the phosphodiester bonds while the polar choline headgroup diffuses into the surrounding medium. Furthermore, Figure 2b demonstrates that changing the PLD concentration relative to the LNP substrate tunes the rate of conversion. PLD concentrations were chosen for a fixed substrate concentration to enable visualization of the conversion time-course in a single experimental run (≈1−2 h). The change in the normalized choline peak signal can be fitted using a first order approximation (Figure 2b) but the determination of Michaelis−Menten parameters is not possible as the PLD concentration is high relative to the substrate that is, DOPC concentration. Therefore, our analysis focused solely on the time taken to convert 50% of the substrate.

The reaction can be visualized as the direct time-course (Figure 2b; Figure S2, Supporting Information) as a function of time with respect to control formulations (Figure 2d) where there is an observable population shift from the substrate-rich DOPC to the reaction product DOPA controlled by the ratio of PLD to DOPC substrate. While the Raman feature of the resulting membrane bound DOPA is less intense, this peak can be consistently measured versus LNP cholesterol in control formulations and as the end product of the enzymatic conversion (Figures S3 and S4, Supporting Information). The enzymatic activity on the DOPC substrate within the LNPs was confirmed with active and inactivated PLD for control formulations with and without substrate and product (Figure S5, Supporting Information), which were supported by statistical analysis of LNP populations (see Supporting Information). The differing PLD time-course is further quantified via the half-life for DOPC conversion (Figure 2c) indicating the nonlinear relationship between enzyme concentrations acting upon the same initial LNP substrates. The Raman spectra are sensitive to the specific compositions of the LNPs and integrate the measured signal for the entire particle trapped within the laser focus; the endpoints obtained indicate the complete conversion of DOPC. Hence, for this formulation, the entire surface of the lipid bilayer within the LNP is accessible to PLD, implying access to an LNP’s entire water channel network. PLD bound on the LNPs was not detected in the SPARTA spectra amidst the lipid signals. This could be due to the transient nature of PLD binding via a hopping mechanism^[39]^ or a small number of enzymes bound to individual LNPs within the SPARTA trapping volume. The LNP structure with accessible water channels, enables free diffusion from solution into and out of the LNPs complicating direct detection of PLD binding (except via composition analysis).

The direct time-course measurements for LNP enzymatic conversion via SPARTA are amenable to kinetic analysis. The dynamics observed herein indicate a potential lagging reaction. This behavior could be explained by activation or autocatalyzation processes, constituting avenues for further investigation. Previous studies have reported that PLD activity is impacted by the presence of the DOPA reaction product: the change in membrane charge and enzyme electrostatic interaction may increase reaction kinetics.^[17]^ Furthermore, the LNPs’ native pore sizes may be the same size or smaller than the size of individual enzymes. However, enzymatic cleavage of the polar headgroup alters the lipid packing, which may allow cascading enzyme influx to increase the reaction kinetics. To interrogate these processes, and the direct relationship between the composition and structure of these bioactive LNPs, we performed thorough structural characterization using SAXS and SANS of the formulations.

### 2.2 Small-Angle Scattering Reveals Different Morphologies in LNPs Produced by Bottom-Up and Enzyme-Induced Lipid Composition Variations

Structural tuning of size and morphology in lipid phases has been demonstrated extensively in bulk and to some extent in LNP format.^[27]^ However, there is less information on enzyme-mediated composition tuning (via phospholipase activity). Here we present a comprehensive SAXS/SANS analysis of morphological changes as a result of LNP lipid composition tuning induced via PLD activity or bottom-up approaches.

#### 2.2.1 Composition-Mediated Morphological Analysis of LNPs

Small-angle X-ray scattering characterization was performed on bulk lipid mixtures with compositions MO:CHOL:DOPC:DOPA *55:30:15-X:X* mol% + 2.5 wt% F127, where *X* = 0, 1.25, 7.5, 15, at 25 and 37 °C (Figure S6, Supporting Information). At *X* = 0, 25 °C, the sample exhibited predominantly bicontinuous cubic morphology (Im3m) with some evidence of inverted hexagonal (H*_II_*) coexistence and cholesterol crystals. With increased mol% of DOPA, the cubic lattice swells, which increases the water channel diameter from 8.1 to 14.2 nm, after which a phase change occurs to a stacked bilayer morphology that is lamellar phase. This is in good agreement with previous observations of swelling cubic phases in bulk and LNP formats via electrostatic interactions.^[24,25]^ The PLD enzyme has dimensions of ≈6 × 4.8 nm^[40]^ and therefore it is feasible that it can be accommodated within the Im3m water channels.

**Figure 3:**
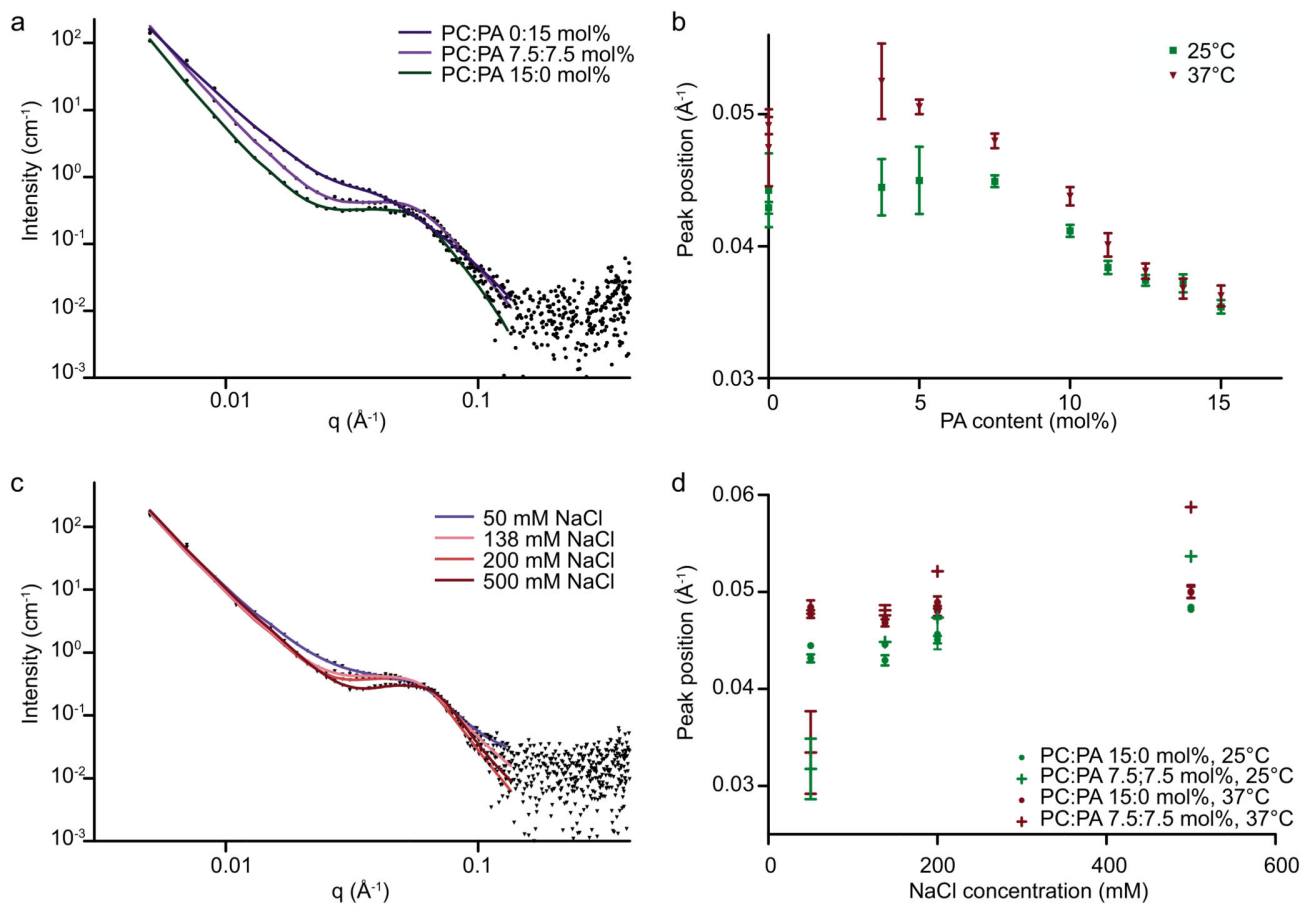
a,b) SANS data for LNPs of composition MO:CHOL:DOPC:DOPA 55:30:15-*X:X* mol% where *X* = 0, 3.75, 5, 7.5, 10, 11.25, 12.5, 13.75, and 15 mol% formulated with 2.5 wt% F127 in 138 × 10^−3^ M NaCl where (a) shows example fits of SANS data at 25 °C for samples where *X* = 0, 7.5, 15 mol%. b) Broad peak positions as a function of mol% DOPA. c,d) SANS data for LNPs of composition MO:CHOL:DOPC:DOPA 55:30:15-*X:X* mol% where *X* = 0, 7.5 mol% formulated with 2.5 wt% F127 in 138 × 10^−3^ m NaCl where (c) shows example fits of SANS data at 25 °C for samples where *X* = 7.5 mol%. d) Broad peak positions as a function of NaCl concentration. Individual points represent an independent measurement with the fitting error.

Small-angle neutron scattering was used to confirm the morphology of LNPs with identical lipid composition to the bulk samples and demonstrate electrostatic tuning of the LNP structure via anionic lipid content, NaCl concentration and temperature (Figure 3; Figures S7 and S8, Supporting Information). Dynamic light scattering showed stable LNPs across all conditions (Figure S8, Supporting Information).

The Bragg peaks visible in the SAXS data (bulk Figure S6, Supporting Information, LNP Figure S9, Supporting Information) are not visible in the SANS data, likely due to the reduced resolution from, for example, differing fluxes and sample concentrations. However, a broad, temperaturedependent SANS scattering was detected in the size region occupied by the Bragg peaks in the SAXS data (Figure S7, Supporting Information). Therefore, to compare the structure sizes detected between SAXS and SANS, the broad peak position extracted from the SANS data fits was used as an indication of internal lattice size (Im3m). For *X* = 15 mol%the statistically significant differences observed showed good agreement with the bulk SAXS data at 25, 37 and 45 °C (Figure S7 and Table S15, Supporting Information). Therefore, it can be assumed that broad peak position is indicative of the Im3m lattice parameter.

Between 0 and 15 mol% DOPA, the LNP water channel diameter increases from ≤5.3 to 7.2 nm at 25 °C indicating a net swelling of the structure with increasing mol% anionic charge (Table S8, Supporting Information). This trend was repeated at 37 °C, where the water channel diameter increases from ≤4.5 to 7.0 nm (Table S9, Supporting Information). At 37 °C, there was also evidence of a coexisting inverted hexagonal H_*II*_ phase characterized by a peak at *q* = 0.1 Å^−1^. These observations are in agreement with the bulk data trends and demonstrate that SANS data models can be applied to internal morphology characterization of LNPs.

To further decouple whether the swelling of the water channel diameter could be attributed to electrostatic swelling effects or alterations in the lipid packing, samples containing DOPC:DOPA 15:0 mol% and 7.5:7.5 mol% were characterized as a function of NaCl concentration (Figure S8, Supporting Information). At 25 °C, the LNP containing 0 mol% DOPC that is, neutrally charged, showed a decrease in the water channel diameter from 5.0/5.3 nm at 50 × 10^−3^ M NaCl to 4.3/4.4 nm at 500 × 10^−3^ M NaCl. However, the LNP containing 7.5:7.5 mol% DOPC:DOPA that is anionic lipid, showed a decrease in the water channel diameter from 8.5 to 3.5 nm. Similar trends were observed at 37 °C. The addition of NaCl to a neutral LNP does reduce the water channel diameter by a maximum of 1 nm over the given NaCl range, however, the decrease in water channel diameter in an LNP containing DOPA is over 5 times this value, therefore, electrostatics are dominating the water channel diameter of the Im3m phase.

In this analysis, the focus is specifically on the broad peak position, however it should be noted that the SANS characterization also facilitates deeper analytical analysis. Further discussion of additional parameters is detailed in the Supporting Information. For these datasets, the broad peak position analysis is sufficient to demonstrate that the size of the internal LNP structure was tunable via electrostatic interactions using anionic lipid content and NaCl concentration.

#### 2.2.2 PLD-Mediated Morphological Analysis of LNPs

To characterize the effects of PLD-mediated conversion of DOPC to DOPA on LNP morphology, SAXS and SANS were performed on LNPs formulated with 15 mol% DOPC and incubated with active PLD, inactive PLD, or no PLD. Radiation damage was observed in selected SAXS samples and therefore characterization was performed at 15 and 30 min following PLD addition. SAXS data showed a coexisting bicontinuous cubic phase (Im3m) with an inverted hexagonal phase (H*_II_*) before PLD incubation (Figure S9, Supporting Information). After PLD incubation, a broad inverted hexagonal (H_*II*_) phase was observed. SANS data obtained after overnight incubation with PLD also showed a broad scattering component in the (H_*II*_) position. These differ from the bulk composition SAXS data in MQ water where increasing the amount of DOPA led to the formation of a highly swollen lamellar phase.

**Figure 4:**
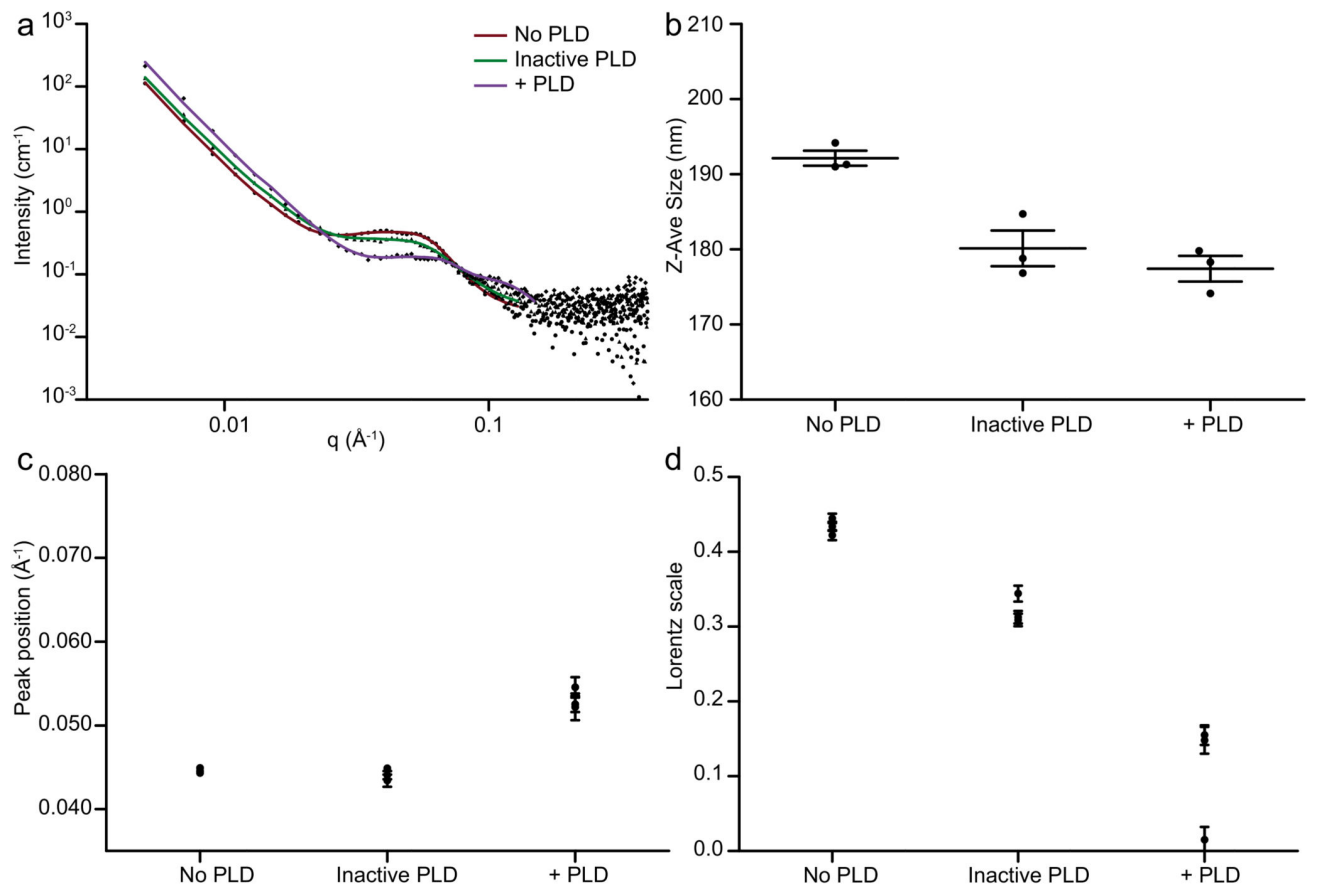
Structural effects of PLD activity on LNPs. SANS measurements of MO:CHOL:DOPC 55:30:15 mol% LNPs incubated with no enzyme (no PLD), boiled enzyme (inactive PLD), or active enzyme (+PLD) for a minimum of 90 min and measured at 25 °C (*N* = 3). a) Representative data and fits. b) Z-Ave size of LNPs (mean ±standard deviation, *N* = 3). c) Broad peak position in LNPs incubated with no PLD, inactive PLD, + PLD. d) Lorentz scale for LNPs no PLD, inactive PLD, + PLD. For (c,d) each datapoint represents an individual fit to a SANS curve where the error bar is the fitting error.

To further characterize changes in structural morphology as a result of PLD activity, SANS and DLS characterizations were performed on LNPs formulated with 15 mol% DOPC and incubated with active PLD for a minimum of 90 min (Figure 4). The extended timescales were chosen to reflect characterization of the equilibrium structures post PLD conversion of DOPC to DOPA. Fitted parameters have been included in the Supporting Information (Table S12, Supporting Information).

DLS measurements showed good sample stability with a PDI below 0.2 for all samples. LNPs incubated with inactive PLD or without PLD exhibited a mean water channel diameter of 5.1 and 5.0 nm, respectively. Incubation with PLD reduced the mean water channel diameter to 3.6 nm. The Lorentz scale, an indication of the broad peak intensity, shows a mean reduction from 0.43 to 0.32 post inactive PLD addition and from 0.43 to 0.11 post active PLD addition. A reduction in the Lorentz scale could indicate a reduction of the internal LNP order either via addition of inactive PLD in glycerol or active PLD in glycerol. To confirm the morphology of LNPs after incubation with PLD, Cryo-TEM was performed to image the LNPs after incubation with PLD (Figure S10, Supporting Information). These images correspond well with the structural data indicating a reduction in the LNP size and reduced ordering within the LNP. Interestingly, the SANS data of LNP morphology post PLD incubation differ from those predicted by the static model where LNPs were formulated to represent the compositions produced as a result of PLD activity. This indicates that whilst LNP composition can be altered via enzymatic (PLD) activity to produce stable LNPs, the resulting LNP morphology cannot be predicted based on composition considerations alone. In the static SANS characterization data we do not account for PLD binding, PLD entrapment within the LNP, or an uneven distribution of lipids across the LNP. From the SPARTA timescales, 50% conversion of DOPC to DOPA has already occurred within 10 min and full conversion of DOPC to DOPA follows. This indicates that PLD has access to the entire DOPC substrate but not necessarily that it is all accessed simultaneously. At any timepoint, PLD-mediated conversion of DOPC to DOPA can occur on the LNP lipid membrane, leading to structural rearrangement. This has particular consequences for pharmaceutical design and delivery of bioactive molecules via enzymatic cleavage where enzyme-mediated processes could be used to produce LNPs with compositions and structures not accessible using bottom-up approaches.

### 2.3 Membrane Curvature Mediates PLD Activity for Lipid Nanoparticles

Highly curved lipid assemblies within cells are associated with organelles including the endoplasmic reticulum, Golgi apparatus, and mitochondria. This conformation has significant advantages over planar structures including higher membrane surface area to volume ratios, variations in membrane stress, and increased hydrophobic and membrane protein loading capacities. Here we use LNPs and vesicles to show that membrane curvature likely plays an important role in the regulation of PLD activity.

**Figure 5:**
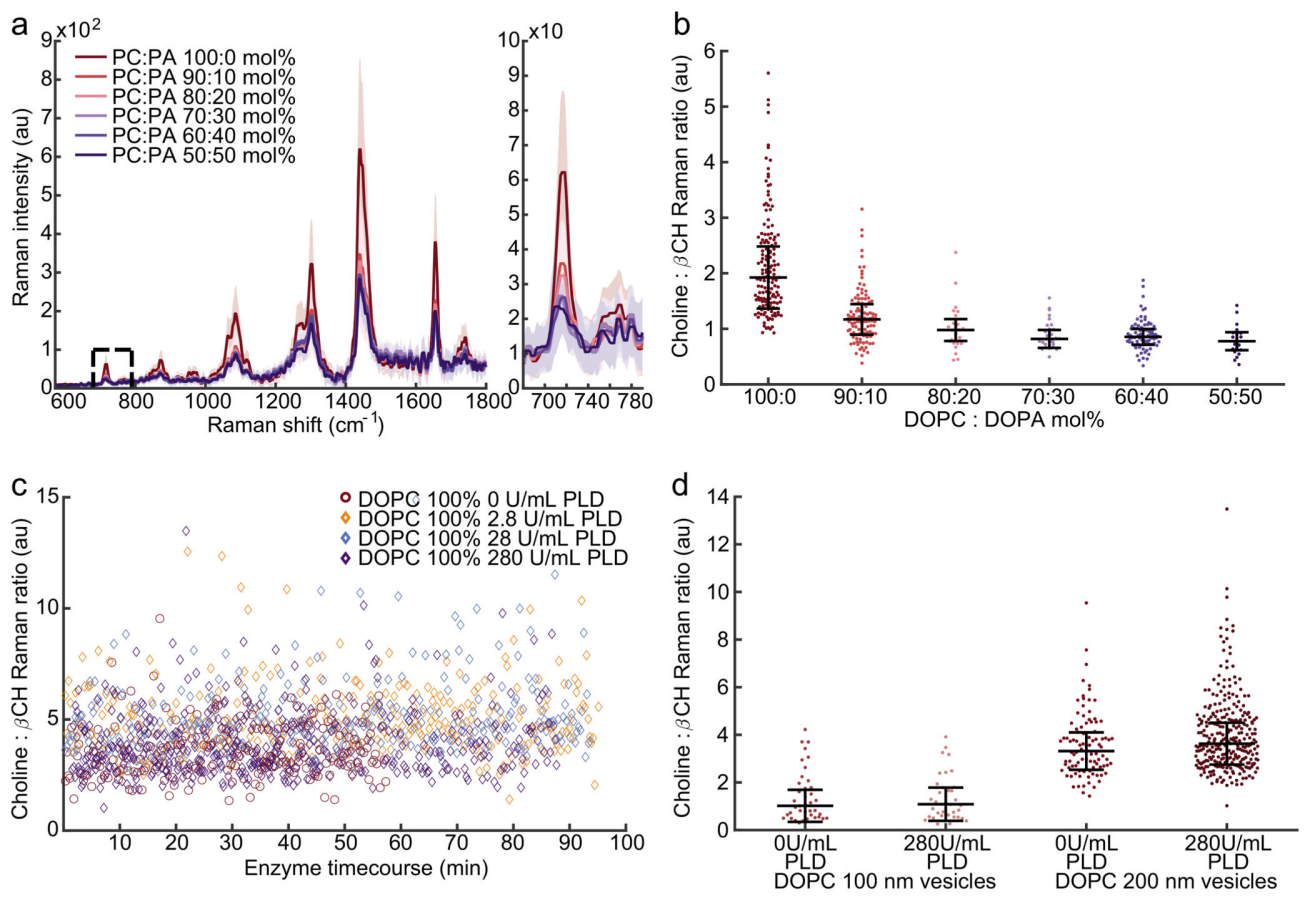
Effects of membrane curvature: PLD activity on extruded DOPC vesicles at 25 °C. a) SPARTA acquired fingerprint Raman spectra from DOPC:DOPA extruded vesicle formulations of varying molar ratios (DOPC:DOPA 100:0, 90:10, 80:20, 70:30, 60:40, and 50:50 mol%) with magnified choline feature region at 718 cm^−1^ (mean ± standard deviation, *n* ≥ 20). b) Choline to *β*CH ratio for distinct vesicle formulations varying DOPC:DOPA mol% ratio (median ± IQR, *n* ≥ 20). c) SPARTA time-course data of PLD activity on 100 nm extruded vesicles with 2.8, 28.0, and 280 U mL^−1^ enzyme. d) Choline to *β*CH ratio (median ± IQR, *n* ≥ 35) for individually trapped 100 and 200 nm extruded vesicle LNPs, neat and with the addition of 280 U mL^−1^ PLD.

Implementing the SPARTA protocols used for LNPs, the ratio of substrate choline to product *β*CH (718/990 cm^−1^) indicates a subtle but detectable, significant difference between vesicle formulations (Figure 5a,b; Figure S4, Supporting Information). Due to vesicle stability, formulations below 50% substrate could not be made. Unlike for LNPs that demonstrated dramatic alterations with enzymatic conversion (Figure 2b), vesicles of comparable substrate concentration displayed no change over the same time-course for matched concentrations of enzyme (Figure 5c). Utilizing different sized vesicles of the same compositions further yielded no significant change in apparent PLD activity (100 and 200 nm vesicles with and without PLD, Figure 5d). Raman spectra of the lipid films comprising pure DOPC and DOPA substrates and extruded 200 nm 100% DOPC vesicles confirmed the characteristic peaks utilized for LNP ratiometric analysis (Figure S11a,b, Supporting Information). To investigate the influence of cholesterol on PLD activity, and to recapitulate the LNP formulations, vesicle membrane assemblies with 70:30 mol% DOPC:cholesterol were measured (Figure S12, Supporting Information) and demonstrated no activity over the same time-course as the LNPs. The signals acquired from vesicles incubated with and without PLD indicate vastly different PLD activity between vesicles and LNPs containing identical amounts of substrate (i.e., DOPC molecules in each sample) for a constant PLD concentration. In order to verify the observed differences between the reaction time-courses of vesicles and LNPs, a fluorescence cascade assay was utilized to monitor PLD activity.

**Figure 6:**
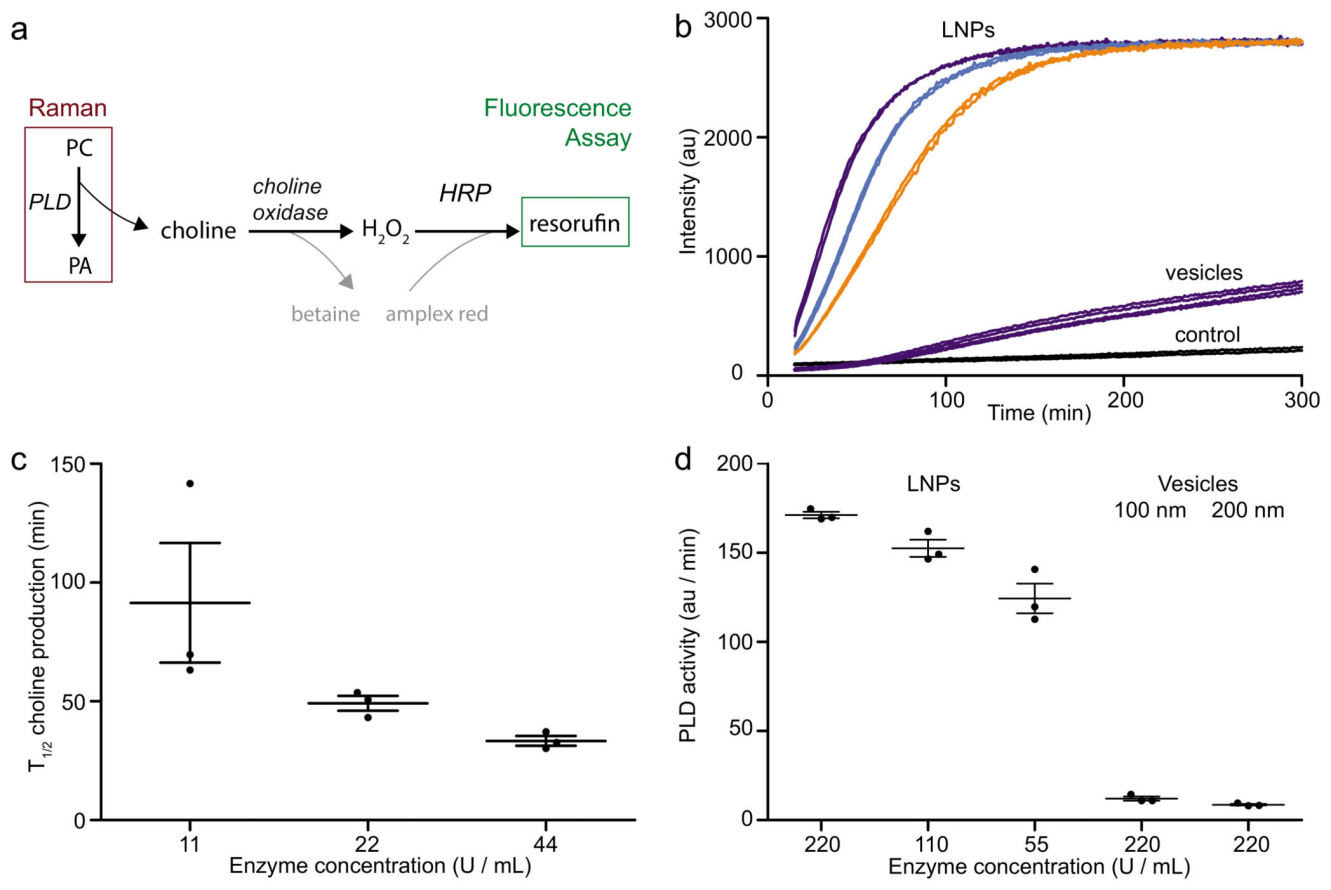
Fluorescent verification of difference in DOPC to DOPA conversion rate between LNP and vesicle samples. a) Reaction schematic. b) Representative fluorescence data at optimized concentrations for LNPs incubated with 11 (orange), 22 (blue), and 44 (purple) U mL^−1^ and 100 and 200 nm vesicles incubated with (purple) 44 U mL^−1^ for 300 min. c) Time to half conversion of DOPC to DOPA for LNP samples (*N* = 3, mean ± standard error, each datapoint is the average of 2-3 wells). d) Comparison of rates between LNPs and vesicles for the conversion of DOPC to DOPA at equal DOPC conversion points (*N* = 3, mean ± standard error).

The fluorescence assay utilizes an enzyme cascade to detect the choline released when DOPC is cleaved by PLD (schematic, Figure 6a). Therefore the rate of fluorescence increase encompasses the rates of several enzymes within the cascade. To enable a more direct comparison with the SPARTA data, we therefore analyzed the time to convert a fixed level of substrate. The assay was optimized to ensure that the maximum fluorescence was below the point of saturation and LNP and vesicle concentrations adjusted to ensure the DOPC concentration was identical in both systems. Whilst exact concentrations of PLD and DOPC differ from the SPARTA measurements, the PLD:DOPC ratio is consistent between the assays. Representative data is displayed in Figure 6b. The maximum fluorescence obtained was linearly proportional to the amount of DOPC substrate present in the sample (Figure S13b, Supporting Information). This was used to compare PLD activities at a linear point in the fluorescence curve. For LNPs with 0 mol% DOPC, fluorescence was stable over the assay time-course. LNPs were measured with three different PLD concentrations (11, 22, 44 U mL^−1^) and vesicles using the maximum PLD concentration only. Representative traces and DLS data are included in the Supporting Information (Figure S14, Supporting Information).

For LNP samples, the time required to reach half the maximum fluorescence (*T*_1_/_2_) that is, to convert half of the DOPC to DOPA was calculated (Figure 6c). Results show a nonlinear concentration dependence for PLD activity: at higher PLD concentrations, DOPC to DOPA conversion is faster which agrees with the SPARTA data. The mean (*T*_1_/_2_) are approximately twice of those calculated using SPARTA, implying that the conversion of choline to resorufin lengthens the observed fluorescence timescales.

At the point of 50% DOPC conversion (half the maximum fluorescence), linear fits to the fluorescence data were performed to calculate PLD activity (Figure S13d, Supporting Information). Under optimized assay conditions, 50% conversion of DOPC in LNPs occurs within the first 100 min, however, for the vesicle system, the 50% conversion of DOPC occurs after 24 h. The LNP and vesicle systems both tend toward the same maximum fluorescence, indicating full conversion of DOPC to DOPA. For vesicles, where PLD does not have access to the inner membrane leaflet, flip-flop of lipid across the different membrane must therefore occur on these timescales. During long PLD incubations, evaporation from the wellplate and enzyme stability (PLD, choline oxidase, HRP) are likely to influence the observed fluorescence. Therefore, the assay was also performed at 5× the lipid concentration of Figure 6b,c and the PLD activity calculated from linear fits (Figure 6d). Under these conditions, the maximum fluorescence is saturated and the midpoint corresponds to ≈1/10 of DOPC conversion. For comparison, the extracted rates for both lipid concentrations are included in the Figure S13c,d, Supporting Information. In both cases, the PLD concentration significantly impacts the rate of choline production from LNPs and the rate of PLD activity in vesicles compared to LNPs is more than an order of magnitude lower. It is possible that the DOPC substrate availability may differ in LNPs and vesicles. In the vesicle bilayer, only 50% of the lipid is available on the outer membrane leaflet and therefore full conversion of DOPC may become limited by lipid flip-flop from the inner to outer membrane leaflets. In practice, there will be a subpopulation of non-unilamellar vesicles and therefore the available DOPC may be even lower, but is unlikely to be as low as 1/10. Given that the enzyme to substrate ratio that is, PLD:DOPC ratio is identical in both cases, this implies that the LNP structure facilitates increased PLD activity. Specifically, PLD activity is increased by increasing the curvature of the lipid membrane. This is supported by the observation that the rate of PLD activity is greater in 100 nm vesicles compared to 200 nm vesicles (12.1 ± 2.0 compared to 8.6 ±0.8 au min^−1^). Differences in PLD activity in different lipid systems have been observed previously in both vesicles^[14]^ and supported lipid bilayers,^[17]^ however this is to our knowledge the only comparison of PLD activity in LNPs and vesicles. This difference in PLD activity between LNPs and vesicles implies that the lipid membrane morphology impacts PLD activity. For the LNPs and vesicles studied, the primary structural difference is the internal lipid membrane with bicontinuous cubic structure present in the LNPs whilst for the vesicles there is an aqueous core. For these differences to impact PLD activity, we infer that PLD has some access to the internal LNP lipid membrane. If the LNP interaction with PLD was solely via the LNP surface, this would be more similar to the PLD - vesicle interaction and would also be limited by diffusion of DOPC to the surface. Therefore, if PLD was unable to access the LNP internal lipid membrane, we expect that the PLD rates observed may resemble the vesicle rates where PLD access is limited to the outer vesicle surface, instead of the order of magnitude difference in PLD activity observed between vesicles and LNPs.

## 3 Conclusions

Combining automated single-particle composition analysis and small-angle scattering provides unprecedented insight into the capacity for LNP structure to drive PLD activity. The SPARTA system enables sensitive detection of LNP lipid composition variations and establishes a platform to quantify LNP formulation homogeneity, dynamics, and subpopulation analysis. Through automated time-course analysis, SPARTA enabled direct visualization of complete PLD-mediated conversion of the DOPC substrate contained within LNPs. This generated LNPs containing negatively charged lipid and revealed full penetration and enzyme access to the entire complex membrane of this LNP population. Direct comparison with vesicle assemblies formulated with equivalent amounts of DOPC substrates indicated PLD activity an order of magnitude higher on LNPs. Coupling LNP composition variations to structure through small-angle scattering (SANS/SAXS), our multiscale analysis reveals dynamic phospholipase-mediated alteration of LNP lipid composition that is directly influenced by controlled tuning of lipid membrane structure. Whilst the cellular environment will introduce complexities (such as protein corona formation) to LNP-phospholipase interactions, this specific interaction is particularly relevant for breast cancer where PLD is overexpressed and has potential for the dynamic generation of anionic LNPs in response to disease. In future, enzyme responsive LNPs could generate new mechanisms for LNP payload delivery. This work demonstrates a broad opportunity to challenge the bottleneck in LNP rational design: to utilize dynamic composition analysis to elucidate structure−function relationships and to probe the interactions of LNP nanocarriers with intracellular trafficking mediators. Nanoscale, single-particle characterization of LNP constructs holds great potential to enable the development of LNP solutions for environmentally responsive delivery in human diseases with altered PLD expression and the optimization of the functional behaviors of LNP formulations as dynamic nanocarriers for delivery of RNA and other novel therapeutics.

## 4 Experimental Section

### PLD Enzyme

PLD from *Streptomyces chromofuscus* was purchased from Sigma Aldrich. The enzyme was supplied in glycerol with a specified activity (U mL^−1^) and diluted appropriately for the relevant experiments in the required solvents to maintain a constant PLD:DOPC (U mg^−1^ DOPC) ratio across the techniques at the maximum concentration. For control experiments, aliquots of enzyme were inactivated via thermal denaturing (90 °C for three cycles).

### Lipid Film Preparation

1-Oleoyl-rac-glycerol and Pluronic F127 were purchased from Sigma Aldrich. Additional lipids (ovine cholesterol (CHOL), 1,2-dioleoyl-sn-glycero-3-phosphocholine (DOPC) and 1,2-dioleoyl-sn-glycero-3-phosphate (sodium salt) (DOPA)) were purchased from Avanti Polar Lipids. All lipids were used without further purification and placed on a lyophilser and dried for a minimum of 24 h before usage. They were subsequently weighed individually into glass vials and dissolved in chloroform to prepare lipid stock solutions of up to a maximum concentration of 20 mg mL^−1^. Lipid films were prepared in glass vials using Hamilton syringes to transfer the appropriate volumes of lipid stock solutions to obtain the desired molar ratios (55 mol% MO, 30 mol% CHOL, varying ratios of DOPA and DOPC (DOPC +DOPA mol%=15) which were then mixed and subsequently dried under a stream of nitrogen and placed overnight on a lyophilizer. Films were stored under nitrogen at −20 °C until required.

### Lipid Nanoparticle Formulation

LNPs were formulated by defrosting the lipid film to room temperature followed by hydration in PBS (+Ca, +Mg) to a concentration of 19 mg mL^−1^. PBS (+Ca, +Mg), (Gibco Life Technologies) was purchased from Sigma Aldrich. Films were freeze thaw cycled a minimum of 10 times between 70 and −79 °C. F127 was dissolved in PBS (+Ca, +Mg) to a concentration of 1.02 mg mL^−1^ and added to the freeze thawed film immediately prior to sonication. The final F127 concentration was 2.5 wt% in all formulations. Samples were sonicated using a Sonics VibraCell VCX 500 tip sonicator using pulsed mode (1 s on, 2 s off) for 2 min and 30 s total sonication time. Suspensions were then centrifuged at 1380 rcf for 10 min at room temperature to remove any tip residue and the supernatant used for experiments. LNP formulations were stored for a maximum of 2 days at room temperature prior to all experiments. Autohydrolysis of the monoolein component in the LNPs is expected to be negligible over the course of 1 week^[41,42]^ and was not observed in any of the control formulation measurements in the absence of PLD (Figure S5, Supporting Information). For SANS measurements, LNPs were diluted to a final lipid concentration of 3.2 mg mL^−1^ in deuterated PBS whilst for SAXS measurements LNPs were formulated at 52 mg mL^−1^.

### Vesicle Formulation

Vesicles were formulated by defrosting the lipid film and hydrating in PBS (+Ca, +Mg) to a DOPC concentration of 5.3 mg mL^−1^ and left to hydrate at room temperature for 30 min. Samples were then vortexed five times for 30 s each time at the maximum vortex speed and extruded using a Hamilton syringe and 100 or 200 nm polycarbonate filter from Avanti Polar Lipids. Vesicle samples were used on the same day as prepared for experiments.

### SPARTA Raman Microspectroscopy System

As previously described, briefly, confocal Raman spectral acquisition was performed on a custom-built laser trapping Raman spectroscopy system.^[32]^ The light source used was a 785 nm laser (Toptica XTRA II) with a 63× 1.0 numerical aperture (NA) water immersion microscope objective lens (W Plan-Apochromat, Zeiss, Oberkochen, Germany). The epicollected scattered light was focused into a 100 μm fibre with a 600 groove mm^−1^ grating spectrograph (UHTS 300, WITec, Ulm, Germany) and spectra were acquired using a thermoelectrically cooled back-illuminated CCD camera (iDus DU401-DD, Andor, Belfast, UK) with a spectral resolution of 3 cm^−1^ and 85 mW laser power at the sample. Laser control and measurement evaluation for automated trapping were remotely controlled via a serial connection and custom MATLAB (2018b, The Mathworks, Natick, MA, USA) scripts.

### SPARTA Standard Sample Preparation

For SPARTA, typical analysis of 200 μL of particle solution was required per measured time-course, of which approximately half was routinely recovered, depending on the measurement time. Ideal particle concentrations were determined to be between 1 × 10^10^ to 10^12^ particles per milliliter. Sample solutions were placed on a 22 mm coverslip, affixed to a standard microscopy slide with a drop of phosphate buffered saline (PBS). The sample was placed under the water immersion objective for measurement, noting the time delay from the addition of enzyme to the first recorded measurement when relevant.

### SPARTA Standard Data Analysis

The following preprocessing procedure was applied to all spectra acquired with the SPARTA platform. The spectral center during acquisition was set at 1000 cm^−1^ for standardization and the raw data was truncated to the range of 340−1825 cm^−1^ to omit the excitation signal. Spectra that were adulterated by cosmic spikes or indicated contaminant particle compositions were removed following manual inspection. Instrument spectral response correction was applied based on NIST reflective standard measurements. Spectral background was subtracted via curve fitting (Whitaker filter, (*λ*) = 100 000) and noise smoothed using a Savitzky−Golay filter (7 points, second order). An automated algorithm was then applied to locate the peak center position and intensity using local parabolic fitting to extract spectral features for comparison. Reported spectra are either normalized to peaks for cholesterol (548 cm^−1^ in ring CH_2_ bending mode) or to lipid backbone CH_2_/CH_3_ vibrations (1445 cm^−1^). Subsequent ratiometric, timecourse, and statistical analyses were implemented in MATLAB.

### SANS Experiments

SANS experiments were performed on the Sans2d beamline at ISIS Muon and Neutron Source, Rutherford Appleton Laboratory, Didcot, UK, using a sample changer with Julabo water bath for temperature control, 1 mm path length quartz cuvette cells and sample volumes of 150 μL. LNP samples were prepared in hydrogenous PBS as described previously and subsequently diluted to 3.3 mg mL^−1^ that is 50 μL into 250 μL deuterated PBS (adjusted to the correct NaCl concentration) and loaded into 1 mm Hellma cells for SANS measurements at 25 °C. Deuterated PBS was prepared using a 10× PBS stock (PBS tablets Gibco) in D_2_O and CaCl_2_ (Sigma Aldrich), KCl (Fluka), and MgCl_2_ (Sigma Aldrich) added to match the hydrogenous PBS. Samples were prepared in 83% D_2_O PBS to minimize incoherent scattering from H_2_O and improve the scattering contrast. Scattering measurements were collected for between 5−10 μA proton current. The beamline was configured with *L*1 = *L*2 = 4 m collimation and sampledetector distances to give a scattering vector *Q* = (4*π*/*λ*)sin(*θ*/2) range of 0.005−0.725 Å^−1^, where *θ* is the scattering angle and neutrons of wavelengths *λ* of 1.75−16.5 Å were used simultaneously by time of flight. Data reduction and background subtraction was performed using MantidPlot^[43]^ and scattering data fitted using SasView v 4.2.2. Samples were fitted using a custom plugin model which comprised of a combined broad peak plus Lorentz peak with the scale and A scale parameters fixed to 1 and the remaining parameters left to vary. If samples showed no evidence of peak formation around 0.1 Å^−1^, the B scale parameter was also set to zero. The full list of fit parameters obtained are detailed in the Supporting Information. Broad peak positions were converted to *d* spacings (*a*) using *a* = 2*π*/*q*. Water channel diameters for the Im3m bicontinuous cubic phase were calculated as described previously.^[24]^

### SAXS Experiments

SAXS experiments were performed on the I22 beamline at Diamond Light Source, Didcot, UK.^[44]^ All samples were measured in SAXS/WAXS mode using using Pilatus 2M detectors and camera lengths of 4.28 m (bulk), 4.77 m (LNPs), and energies of 18 keV (bulk), 12.4 keV (LNPs). Lipid films were hydrated and freeze thaw cycled as described previously in either 90 wt% MQ water (bulk samples) or 95 wt% PBS (+Ca, +Mg) (LNP samples) and loaded into polycarbonate capillaries (Spectrum Plastics, US) and measured at 25 °C, 37 °C (bulk), or 37 °C (LNP samples). Radiation damage was observed in PBS hydrated LNP samples after a few minutes of irradiation, so samples were only measured at 37 °C where PLD activity was expected to be highest. SAXS data was analyzed using AXcess, an in house software package.^[45]^ Briefly, the 2D SAXS images were radially integrated to give 1D diffraction patterns. The Bragg peaks were then fitted and indexed using characteristic peak spacings from known lipid structures to extract the *d* spacings. For the inverted hexagonal H_*II*_ phase *d* spacings were converted to lattice parameters as described previously.^[46]^ Water channel diameters for the Im3m bicontinuous cubic phase were calculated as described previously.^[24]^

### Fluorescence Assay

LNPs and vesicles were prepared as described previously. Samples were diluted so that the DOPC concentration (assumed from the lipid film formulations) was identical in the vesicle and LNP experiments. The final DOPC concentration used in the PLD activity assay was either 16.7/83.4 μg mL^−1^. The PLD activity assay was purchased as a kit from Thermo Fisher Scientific (Invitrogen Molecular Probes Amplex Red Phospholipase D Assay Kit) and optimized for these experiments. Under optimized conditions, the lipid substrate in the assay was removed and either LNPs or vesicles were used to replace the substrate. The other assay components were PBS (+Ca, +Mg), 0.13 U mL^−1^ choline oxidase, 1.33 U mL^−1^ horseradish peroxidase and 66.7 × 10^−6^ m amplex red. Experiments were performed using a 96 wellplate and measured on a VarioskanLux platereader in kinetic fluorescence mode with an excitation wavelength of 530 nm, an emission wavelength of 590 nm and a measurement performed every min. In between measurements the plate was subjected to gentle shaking (60 rpm). PLD activity was quantified at PLD:DOPC ratios of 2616, 1308, and 654 U PLD per mg DOPC. This corresponded to PLD assay concentrations of 44, 22, and 11 U PLD per mL for a DOPC concentration of 16.7 μg mL^−1^. PLD activity was quantified using two methods: 1) the time taken for 50% of substrate to product conversion to occur (for a DOPC concentration of 16.7 μg mL^−1^; 2) the rate of fluorescence increase as a function of time via a linear fit with five datapoints around the 50% maximum fluorescence point (for DOPC concentrations of 16.7 or 83.4 μg mL^−1^). It is important to note that these rates are not directly equivalent to the Raman rates as the fluorescence activity assay indicates the time required to produce resorufin within an enzyme cascade that includes choline oxidase and horse radish peroxidase (HRP). Validation of enzymatic assay endpoints was performed by increasing the PLD concentration to 220 U mL^−1^, plotting the max obtained fluorescence as a function of DOPC concentration and fitting a straight line to the calibration data. LNP samples containing 0 mol% DOPC did not result in a positive fluorescence change using the PLD activity assay. Measurements were performed at 25 °C. Time measurements were adjusted to account for the time taken to set up the experiments and begin measurements.

### DLS Characterization

Samples for DLS characterization were diluted 10 μL into 490 μL of PBS (+Ca, +Mg) and measured at 25 °C in low volume cuvettes using a Malvern Zetasizer Nano series (Nano - ZS) DLS. Measurements were performed in triplicate on each sample. The *Z* average size that is, the intensity weighted mean hydrodynamic size and the polydispersity index are reported with representative raw data curves in the Supporting Information.

### Cryo Electron Transmission Microscopy

LNPs (Figure 1) were diluted in PBS (+Ca, +Mg), pipetted onto glow discharged (40 s, 10 mA) Quantifoil R2/2 Cu 200 mesh grids (Electron Microscopy Services), blotted using a Vitrobot markIV (Thermo Fisher Scientific) at 100% humidity, 25 °C, before freezing in liquid ethane. For the Supporting Information, where the LNPs were incubated with PLD, samples were frozen on C flat grids R2/2 and glow discharged for 40 s, 20 mA. All other conditions were constant. Grids were stored in liquid nitrogen prior to Cryo-TEM image acquisition on a 200 kV Talos Arctica (Thermo Fisher Scientific) equipped with a Falcon 2 direct electron detector. Images were recorded with a magnification of either 28k× or 45k×, a defocus ranging from −3 to −6 μm, and an electron dose of 24 e^−^ Å^−2^. Raw images were imported into ImageJ and Adobe for processing and addition of scale bars. Grids were imaged at the CryoEM National Facility, SciLifeLab, Stockholm in collaboration with M. Carroni.

### Statistical Analysis

All statistical analysis performed for this study was implemented in MATLAB. For SPARTA data, please refer to the previous section for details on pre-processing of data (e.g., transformation and normalization). For all other data presented, the processing has been detailed in the relevant experimental sections. Based on samples sizes and goodness-of-fit testing for normally distributed data, the SPARTA data were represented throughout the figures as median ± interquartile range (IQR). All other data sets contained too few data points to calculate IQR and were represented as mean ± standard deviation unless otherwise noted. Each analysis and figure indicate the sample size (*n*) per group for interpretation of the statistical analysis. When possible (i.e., *n* ≥ 4 per group), the Lillifores test was used to assess distribution normality. Data derived from non-normal distributions were compared using the Kruskal− Wallis test in the case of more two than groups or Wilcoxon rank sum test for the difference between medians of two groups (*α* = 0.05). For data groups including *n* ≤ 5 values, one-way analysis of variance (ANOVA) was utilized for differences between groups. Multiple comparisons for significant Kruskal−Wallis and one-way ANOVA tests were assessed using the Tukey−Kramer method (*α* = 0.05, two-tailed) to identify significant group differences. All relevant figure legends include the information on sample size (*n*), and data presentation. For clarity, all specific statistical analysis per group has been presented in the Supporting Information.

## Supplementary Material

Supplementary Materials

## Data Availability

The data that support the findings of this study are openly available in Zenodo at https://doi.org/10.5281/zenodo.6414799. Code is available on request from rdm-enquiries@imperial.ac.uk subject to any restrictions related to IP filing.
