# Supplementary material for: Coupling Lipid Nanoparticle Structure and Automated Single-Particle Composition Analysis to Design Phospholipase-Responsive Nanocarriers
Source: Adv Mater. Author manuscript; Available in PMC 2024 Jan 6. (PMC7615489; doi:10.1002/adma.202200839)
Supplement: Supplementary Materials [file EMS175893-supplement-Supplementary_Materials.pdf]

ADVANCED MATERIALS

Supporting Information

for *Adv. Mater.,* DOI: 10.1002/adma.202200839

Coupling Lipid Nanoparticle Structure and Automated Single-Particle Composition Analysis to Design Phospholipase-Responsive Nanocarriers

*Hanna M. G. Barriga, Isaac J. Pence, Margaret N. Holme, James J. Doutch, Jelle Penders, Valeria Nele, Michael R. Thomas, Marta Carroni, and Molly M. Stevens**

SUPPLEMENTARY INFORMATION

Coupling Lipid Nanoparticle Structure and Automated Single Particle Composition Analysis to Design Phospholipase Responsive Nanocarriers

1.1 Description of experimental pipeline

The experimental pipeline has been outlined schematically in Figure S1. Briefly this involved LNP morphology characterization via DLS, Cryo-TEM, small angle scattering (X ray (SAXS) and neutron (SANS)) and dynamic monitoring of PLD activity via SPARTA and fluorescence measurements.

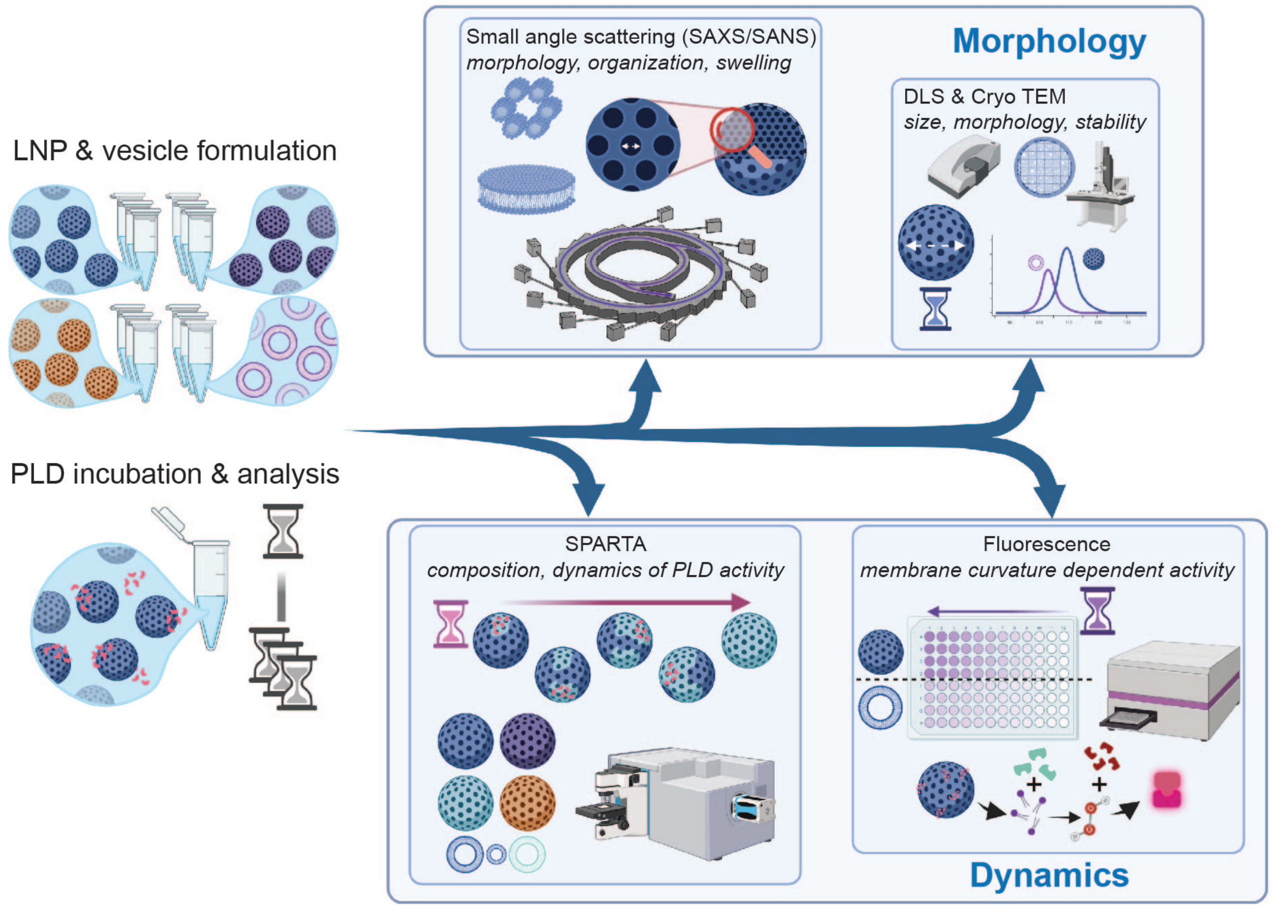

Supplementary Figure 1: Schematic of the experimental pipeline for investigating PLD and LNP interactions. Created with BioRender.com.

1.2 Analysis of LNP SPARTA® data

Enzymatic activity of PLD on individually trapped DOPC-containing LNPs is most clearly analyzed by direct comparison to signals from a constituent lipid that is unaffected by PLD hydrolysis. The selection of the cholesterol ring breathing feature, the peak at 703 cm^{-1}, is a natural choice to normalize particle DOPC content as it does not overlap with other spectral peaks and is in the same spectral region as the peak related to the symmetric stretching vibration of DOPC choline

(tertiary amine) that the PLD cleaves (ν_s $N^+(CH_3)_3$, 718 cm^{-1}). Time course analysis of individual particles also displays predictable and controlled decreases in choline : cholesterol for MO:CHOL:DOPC:DOPA 55:30:15:0 mol% LNPs with rates of activity determined by PLD concentration (Fig. S2A). The reaction proceeds monotonically, however the simple first-order kinetic fitting shows discrepancies from the data. Residual analysis reveals a linear trend in the Lineweaver-Burk plot (log of reaction velocity versus substrate concentration) indicating a lagging reaction that plateaus as the available DOPC substrate is depleted, gradually progressing from choline ratios consistent with neat MO:CHOL:DOPC:DOPA 55:30:15:0 mol% LNPs to total conversion, matching the signals obtained from neat MO:CHOL:DOPC:DOPA 55:30:0:15 mol% LNPs. The same data can be represented as a distribution, while representing time following the enzyme addition as the color axis (Fig. S2B). It is relevant to note that time course change is normalized between DOPC : DOPA 15:0 mol% and 0:15 mol% LNPs which match the endpoints of the enzyme time course. The different concentrations of PLD introduced to the LNP samples cause an observed shift in the distribution of the choline : cholesterol ratio as a function of time. The changes in the apparent spread enzyme activity may be related to the number of enzymes per LNP and may account for the lagging reaction kinetics observed. Within the control group LNPs (DOPC : DOPA 15:0 and 0:15 mol%) the spread in the choline : cholesterol ratio data represents the uncertainty of an individual particle measurement which could be caused by several potential sources including variations in LNP size, LNP composition, or LNP movement within the SPARTA trapping volume. As the DLS sizes for LNP formulations are narrow, further investigation would be required to isolate the source of these differences, however, the variation in choline : cholesterol Raman ratio intensity is $\pm$ 7% of the median signal for individually trapped DOPC : DOPA 15:0 mol% LNPs and $\pm$ 15% from the median signal of individually trapped DOPC : DOPA 0:15 mol% LNPs. The increase in variation for DOPC : DOPA 0:15 mol% LNPs is also a function of the low but present underlying background peak that appears as a shoulder in the absence of the Choline peak at 718 cm^{-1}.The variability in the peak intensity over time within these normalized spectra indicates some heterogeneity between particles. This peak fluctuation likely accounts for the significant differences found between control formulations that lack DOPC both with and without active PLD, however the magnitude of these differences is well below level of signal changes consistent with PLD activity in DOPC containing LNPs. In the absence of PLD, these could be caused by variations in LNP size and lipid composition. However, in the presence of PLD, peak intensity variations may indicate a heterogeneous distribution of PLD across the LNP population or a range of enzymatic activity rates. Further investigations are needed to investigate these mechanisms further.

Supplementary Table 1: Statistical analysis between control LNP formulations with SPARTA. Groups 1 and 2 represent different LNP lipid compositions (mol %). Data from Fig. 1d.

Group 1	Group 2	p-value
MO55 PA00 PC15 Ch30	MO55 PA05 PC10 Ch30	0.0000
MO55 PA00 PC15 Ch30	MO55 PA7.5 PC7.5 Ch30	0.0000
MO55 PA00 PC15 Ch30	MO55 PA15 PC00 Ch30	0.0000
MO55 PA05 PC10 Ch30	MO55 PA7.5 PC7.5 Ch30	0.0000
MO55 PA05 PC10 Ch30	MO55 PA15 PC00 Ch30	0.0000
MO55 PA7.5 PC7.5 Ch30	MO55 PA15 PC00 Ch30	0.0000

While this analysis is presented normalized to a stable lipid component that is both present in this formulation and not altered by enzyme activity, the same trends hold for the ratio of substrate versus product, and enable analysis of DOPA:DOPC systems without cholesterol. LNP structures that do not contain cholesterol cannot be normalized to the cholesterol ring breathing feature in the same way (Fig. S3B). To provide a consistent analysis method between LNP structures with and without constituent cholesterol, the same data was normalized with each trapped LNP Raman spectrum to the CH_2 bending vibration (peak at 1440 cm^{-1}) that originates from the hydrocarbon backbone of the lipid components. As such, it is also largely unaffected by cleaving of headgroups. Direct comparison between Fig. S2A,B and Fig. S2C,D reveals identical trends for time course activity of PLD on LNPs independent of cholesterol signal comparison. It is worth noting that due to the higher peak intensity of CH_2 relative to the cholesterol ring breathing, this significantly alters the ratio of absolute signal intensities, however this has limited impact on the analysis.

As PLD cleaves the headgroup from DOPC and free choline diffuses away, there is a change in the bending and vibrational frequencies of the phosphatidic acid backbone. Hence, observation of a small but consistently increasing CH bending feature (βCH, 990 cm^{-1}) is observed among the trapped particles for control formulations (Fig. S3A) and time course spectra (Fig. S2E,F). As can be seen by comparison of Fig. S4A,B (which have been reproduced from Fig. 2B,C for simplified evaluation) with Fig. S4C,D, both substate degradation and product formation display consistent behavior for single trapped LNPs. The CH bending spectral feature is representative of PA formation following PLD activity however depicts a small time lag relative to choline degradation (Fig. S4B,D). It is worth noting that the peak amplitude of the βCH feature is low and the position falls on the shoulder of another spectral feature, which may potentially limit sensitivity of this feature for analysis and contribute to statistically significant differences in control groups when assessed, even considering the small change in magnitude. Despite this limitation, analysis of this peak is indicative and consistent for PA product formation.

Supplementary Table 2: Statistical analysis between PLD activity on LNPs with SPARTA. Time to reach 50% DOPC conversion as a function of PLD concentration. Data from Fig. 2c and Supp. Fig. 4B.

Group 1	Group 2	p-value
LNP with 70 U/mL PLD	LNP with 140 U/mL PLD	0.0000
LNP with 70 U/mL PLD	LNP with 280 U/mL PLD	0.0000
LNP with 140 U/mL PLD	LNP with 280 U/mL PLD	0.4870

Supplementary Table 3: Statistical analysis between PLD activity on LNPs with SPARTA. Time to reach 50% DOPA formation as a function of PLD concentration. Data from Supp. Fig. 4D.

Group 1	Group 2	p-value
LNP with 70 U/mL PLD	LNP with 140 U/mL PLD	0.0000
LNP with 70 U/mL PLD	LNP with 280 U/mL PLD	0.0000
LNP with 140 U/mL PLD	LNP with 280 U/mL PLD	0.6407

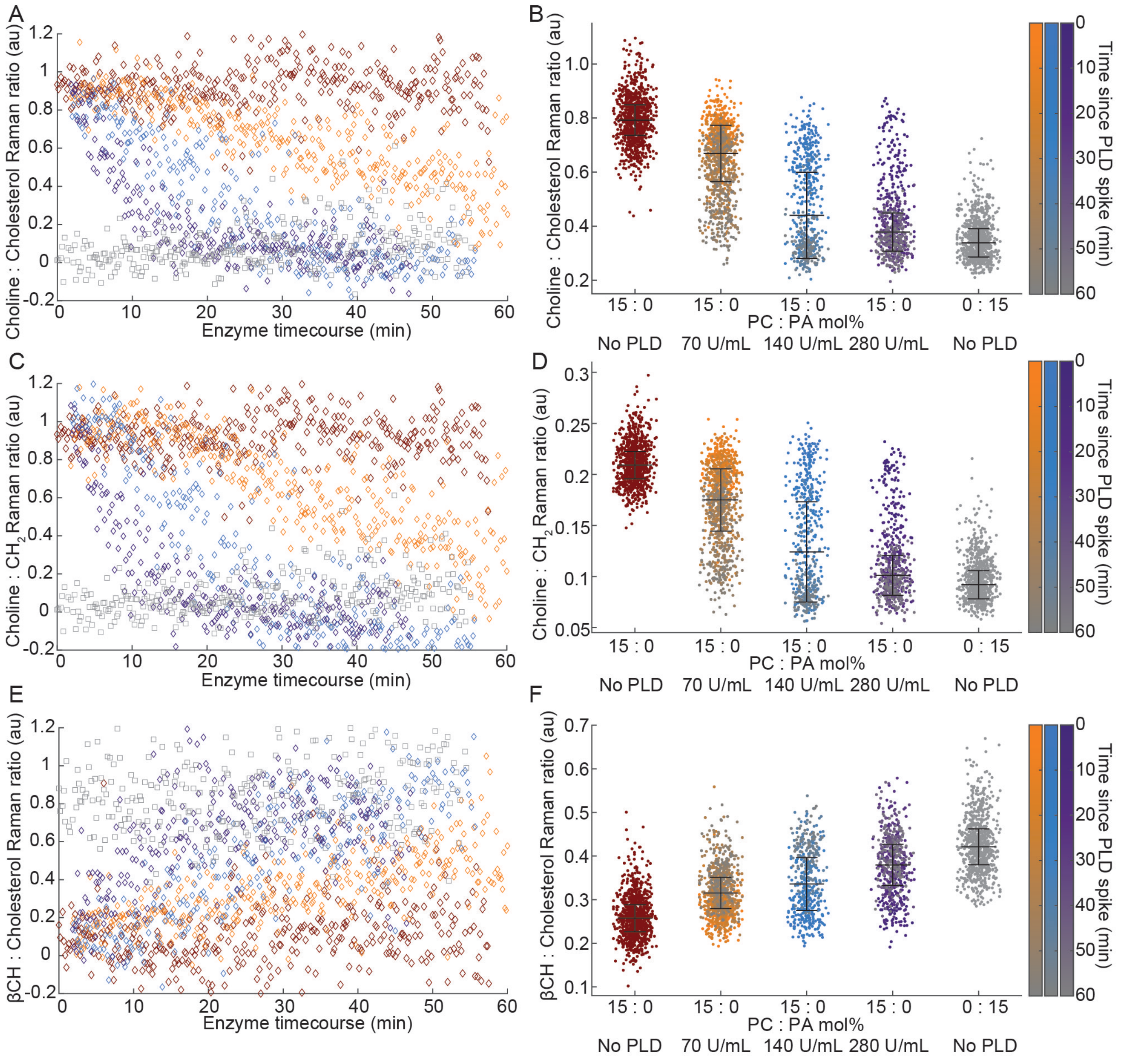

Supplementary Figure 2: Substrate degradation and product formation via phospholipase D degradation of single LNPs as a function of enzyme concentration. Normalized change in individually trapped MO:CHOL:DOPC mol% 55:30:15 LNPs after addition of 280, 140, or 70 U/mL PLD and control LNPs MO:CHOL:DOPC mol% 55:30:15 and 55:30:0 over a 60 minute time course. Consistent behaviour is displayed for time course experiments calculated for (A) DOPC substrate degradation versus cholesterol ring deformation (Choline : cholesterol, 718 cm^{-1}/ 703 cm^{-1}), (C) DOPC substrate degradation versus CH_2 backbone scissoring vibrations (Choline : CH_2, 718 cm^{-1}/ 1440 cm^{-1}), and (E) formed DOPA CH bending versus cholesterol ring deformation (βCH : cholesterol, 990 cm^{-1}/ 703 cm^{-1}). Alternatively, the time course data from A, C & E can be represented as beeswarm plots in B, D, & F, respectively, such that the time of the individual particle trap relative to the enzymatic time course is encoded with color, n $\geq$ 495. Here, the distribution of SPARTA® Raman ratio values from neat MO:CHOL:DOPC mol% 55:30:15 LNPs shifts towards neat MO:CHOL:DOPC mol% 55:30:0 ratio more rapidly with higher PLD enzyme concentration.

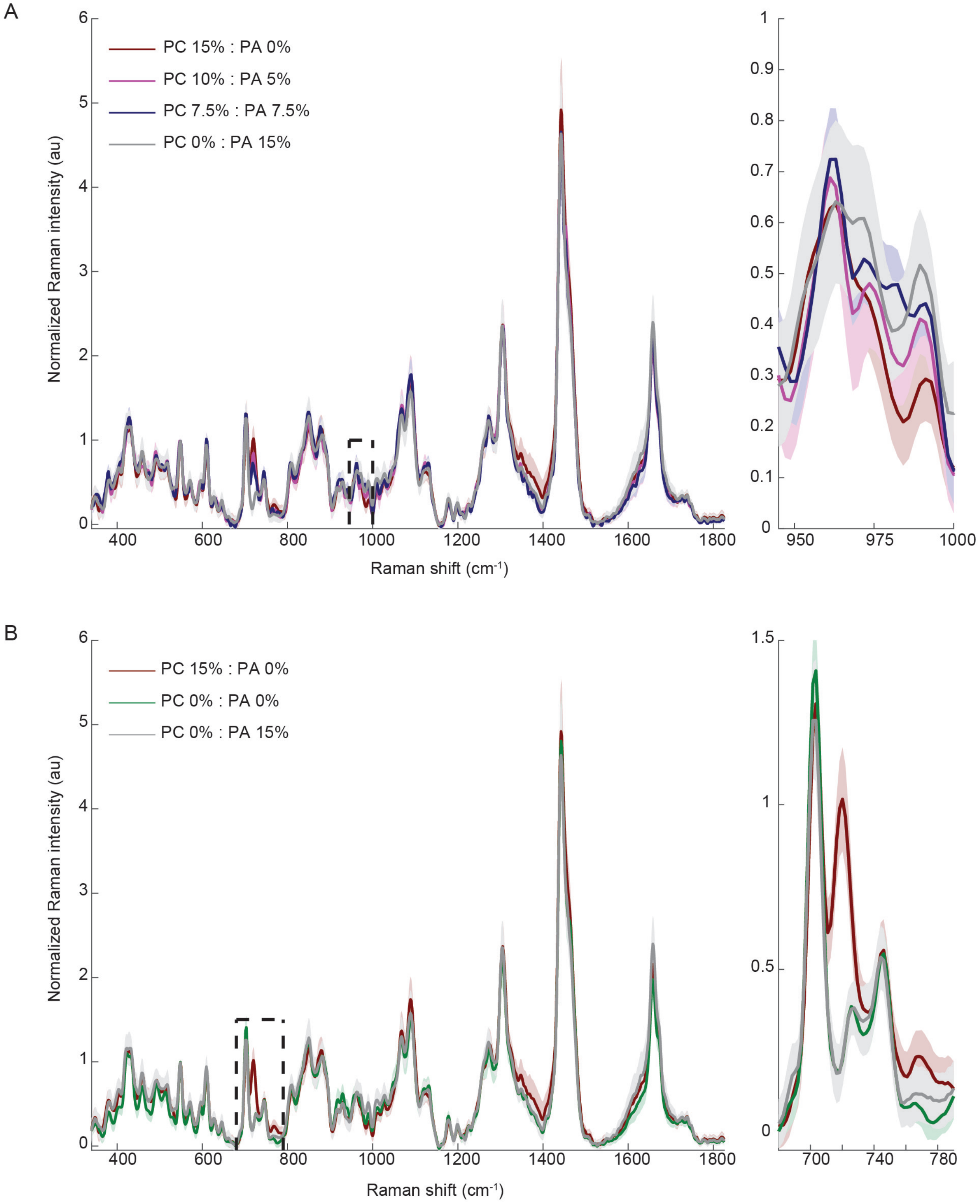

Supplementary Figure 3: Extracted SPARTA® Raman spectra for populations of individually trapped LNPs fabricated with different controlled compositions. (A) MO:CHOL:DOPC:DOPA mol% 55:30:15-X:X, where X = 0, 5, 7.5 & 15, with inset magnified for the βCH spectral feature (990 cm^{-1}) demonstrating controlled differences in DOPA specific feature that corresponds to formulation, n $\geq$ 175. (B) MO:CHOL:DOPC:DOPA mol% 55:30:15:0, 70:30:0:0, & 55:30:0:15, with the inset depicting the magnified region of DOPC Choline symmetric stretching vibration (ν_s $N^+(CH_3)_3$, 718 cm^{-1}) and cholesterol ring breathing (703 cm^{-1}), n $\geq$ 675. All spectra are represented as mean ± standard deviation.

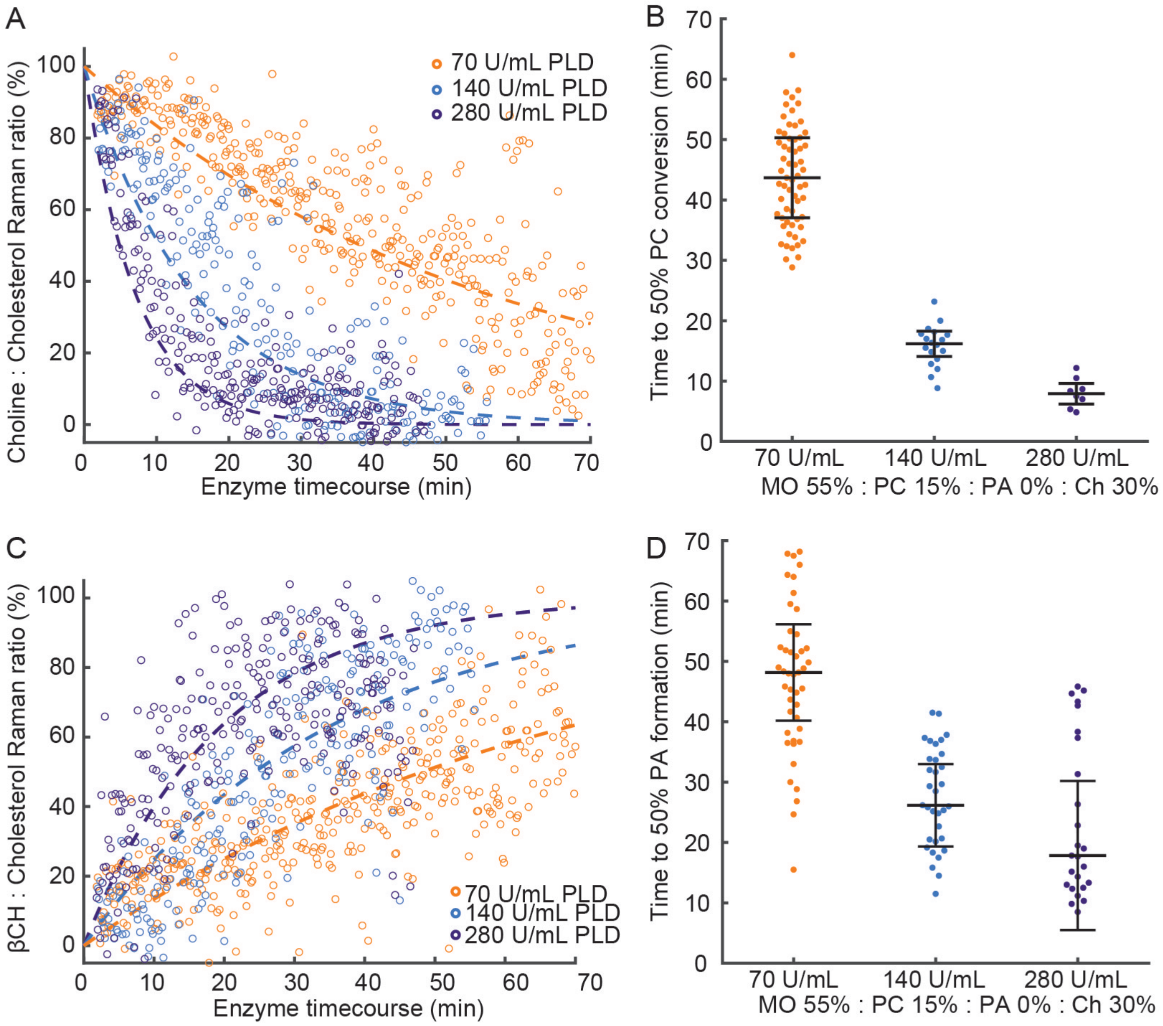

Supplementary Figure 4: Substrate degradation and product formation via phospholipase D degradation of single LNPs as a function of enzyme concentration. Normalized change in individually trapped MO:CHOL:DOPC mol% 55:30:15 LNPs after addition of 280, 140, or 70 U/mL PLD over a 70 minute time course. (A) Normalized DOPC substrate Choline degradation versus cholesterol ring deformation (Choline : cholesterol, 718 cm^{-1}/ 703 cm^{-1}). Reproduced from Fig. 2B for comparison with product formation. (B) Extracted time from enzymatic time course for individually trapped LNPs with 50% ± 5% Choline : cholesterol Raman ratio (718 cm^{-1}/ 703 cm^{-1}) as a function of PLD concentration (median ± IQR, n = 61, 18, & 8, respectively). Reproduced from Fig. 2C for comparison with product formation. (C) Normalized DOPA CH bending versus cholesterol ring deformation (β(CH) : cholesterol, 990 cm^{-1}/ 703 cm^{-1}). Dashed lines indicate first order kinetic approximation for enzyme time course with 280, 140, and 70 U/mL phospholipase D. Represented datapoints indicate extracted control LNP normalized Raman ratio data from individually trapped MO:CHOL:DOPC mol% 55:30:15 LNPs as a function of time since enzyme addition, for N $\geq$ 5 independent time course experiments. (D) Extracted time from enzymatic time course for individually trapped LNPs with 50% ± 5% βCH : cholesterol Raman ratio (990 cm^{-1}/ 703 cm^{-1}) as a function of PLD concentration (median ± IQR, n = 45, 33, & 25, respectively).

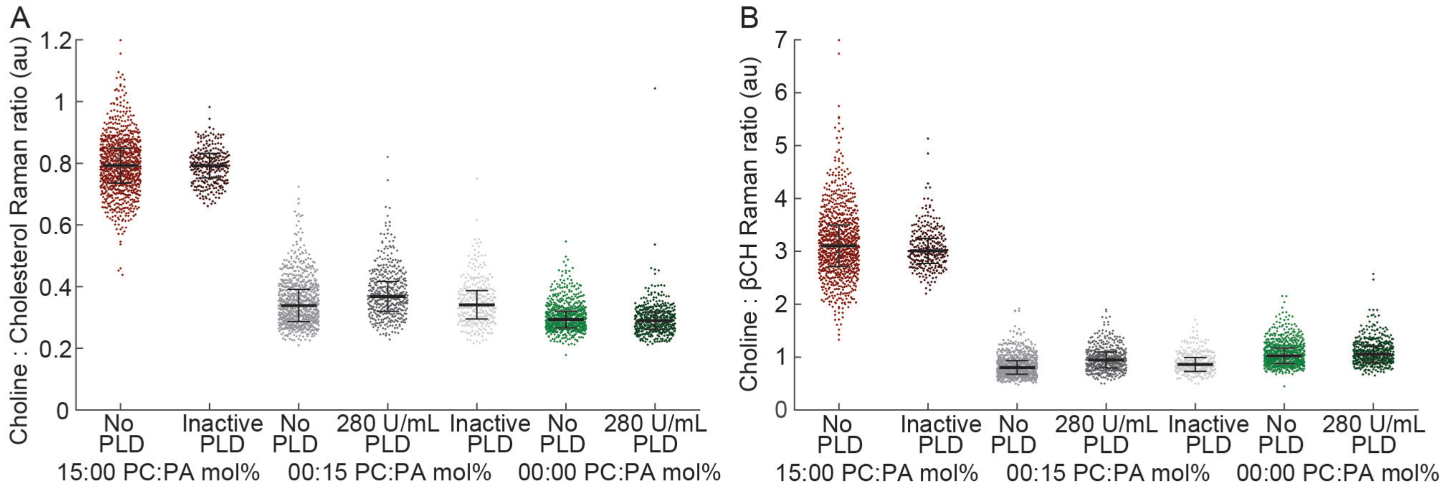

Supplementary Figure 5: Quantification of the (A) cholesterol ring breathing normalized (703 cm^{-1}) and (B) CH bending normalized (990 cm^{-1}) Choline signal (718 cm^{-1}) (median ± IQR, n ≥ 285) of individual particles for control LNP formulations (MO:CHOL:DOPC:DOPA mol% 55:30:15:0, 70:30:0:0, and 55:30:0:15 mol%) as native LNPs and with the addition of active and inactive PLD. (A) is reproduced from 2D for direct comparison with normalization that does not consider cholesterol content. Expected heterogeneity of individual LNPs can be observed while PLD activity is only present for experiments with both active enzyme and LNP formulations containing the DOPC substrate. The consistent performance between the analyses indicates specificity of PLD to activity only for DOPC containing formulations.

Supplementary Table 4: Statistical analysis between PLD activity on control LNP formulations with SPARTA. Data from Fig. 2d and Supp. Fig. 5A.

Formulation	Group 1	Group 2	p-value
MO55 PA00 PC15 Ch30	No PLD	Inactive PLD	0.3918
	No PLD	Active PLD	0.0000
MO55 PA15 PC00 Ch30	No PLD	Inactive PLD	0.9088
	Active PLD	Inactive PLD	0.0000
MO70 PA00 PC00 Ch30	No PLD	Active PLD	0.0058

Supplementary Table 5: Statistical analysis between PLD activity on control LNP formulations with SPARTA. Data from Supp. Fig. 5B.

Formulation	Group 1	Group 2	p-value
MO55 PA00 PC15 Ch30	No PLD	Inactive PLD	0.0653
	No PLD	Active PLD	0.0000
MO55 PA15 PC00 Ch30	No PLD	Inactive PLD	0.0000
	Active PLD	Inactive PLD	0.0002
MO70 PA00 PC00 Ch30	No PLD	Active PLD	0.0358

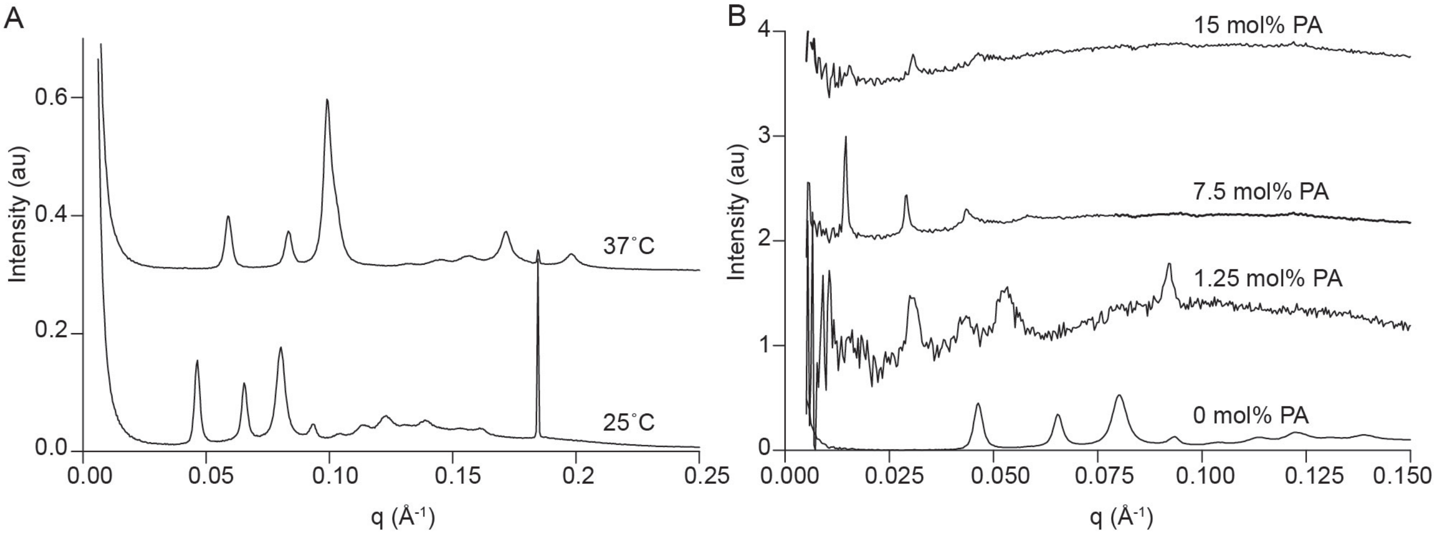

Supplementary Figure 6: (A) SAXS data from a bulk sample of MO:CHOL:DOPC 55:30:15 mol% + 2.5 wt% F127 hydrated in 90 wt% MQ water and measured at 25 °C and 37 °C. (B) SAXS diffraction data from bulk samples of MO:CHOL:DOPC:DOPA at ratios of 55:30:15-X:X mol% where X = 0, 1.25, 7.5, 15 mol% in 90 wt% MQ water and measured at 25 °C.

1.3 Bulk SAXS analysis

SAXS of LNPs requires concentrated samples (e.g. 95 wt% water) which limits the experimental feasibility for costly reagents. The use of phosphate buffers such as PBS in combination with certain lipids e.g. phosphocholine can also lead to radiation damage of the LNPs. To characterise the expected phase morphology of MO:CHOL:DOPC:DOPA + 2.5 wt% F127 mixtures and structural consequences of changing the DOPC:DOPA ratio, we characterized bulk lipid mixtures in MQ water at 25 and 37 °C (Fig. S6). At 25 °C and DOPC mol% = 15 the predominant phase observed was the bicontinuous cubic Im3m phase with lattice parameter 191.8 ± 0.2 Å. At 37 °C the Im3m lattice parameter reduced to 150.6 ± 0.3 Å and a coexisting inverted hexagonal H_{II} phase (lattice parameter 73.3 ± 0.0 Å) was observed. On closer inspection, a weak H_{II} phase (lattice parameter 90.1 ± 0.2 Å) can also be observed at 25 °C in coexistence with the Im3m phase. To preserve the cubic Im3m morphology and maximise sample homogeneity it was decided to study the LNP mixture further at 25 °C. Increasing the mol% of DOPA to DOPC:DOPA mol% = 13.75:1.25 led to a swelling of the Im3m cubic phase (lattice parameter 291.6 ± 1 Å) whilst the coexisting H_{II} phase remained stationary. At 7.5 : 7.5 mol% of DOPC : DOPA, a highly swollen lamellar phase (lattice parameter 433.7 ± 1.7 Å) was observed. This decreased to 413.0 ± 3.5 Å at 15 mol% DOPA. These samples demonstrate that at equilibrium, increasing the amount of anionic charge in the Im3m bicontinuous cubic phase swells the phase i.e. the diameter of the water channels and eventually leads to a phase change to the lamellar phase. This phase behaviour is in agreement with previous literature.[1]

1.4 Composition mediated SANS analysis

SANS data fitting of dispersions (N = 3) containing 15 mol% DOPC at 25 °C were found to have a mean broad peak position of 0.045 ± 0.001 $Å^{-1}$ which equates to a lattice parameter, "a" of 139.2 ± 2.1 Å where $a = 2\pi/q$. Comparisons of SAXS / SANS lattice approximations for X = 15 mol% as a function of temperature are shown in Table 15 and Fig S7.

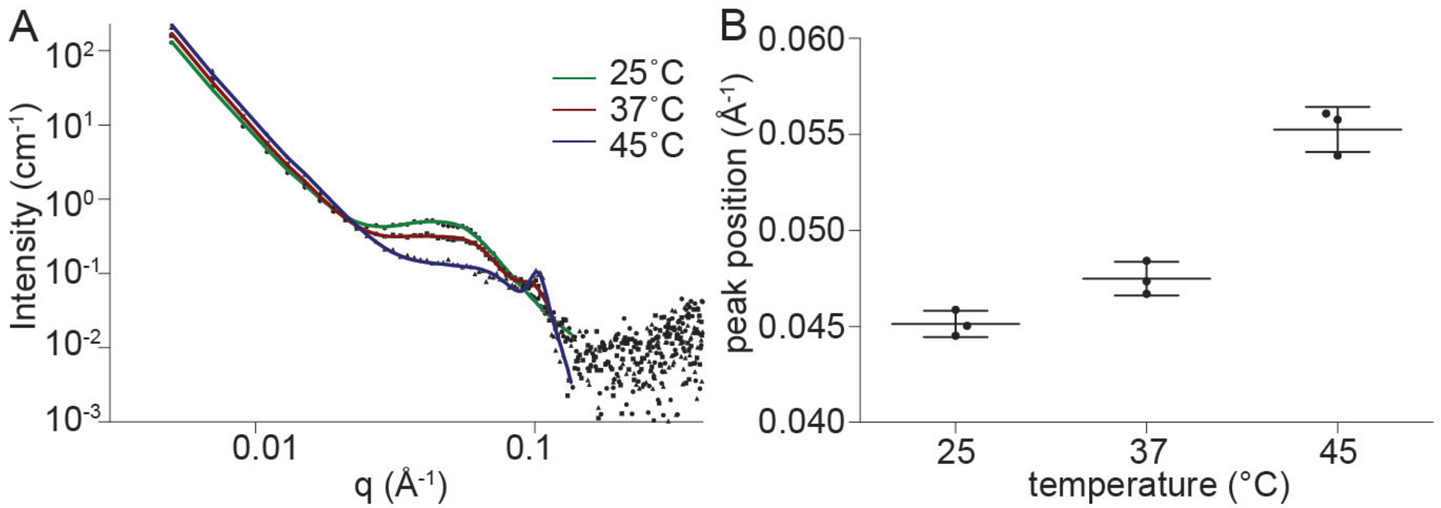

Supplementary Figure 7: SANS data for LNPs of composition MO:CHOL:DOPC 55:30:15 mol% formulated with 2.5 wt% F127 in PBS (A) Example fits of SANS data (B) Broad peak positions as a function of temperature (N = 3, mean and standard deviation).

Briefly, both SAXS and SANS data show a decrease in the lattice parameter with increasing temperature and a growth of the inverted hexagonal H_{II} phase. This enables us to attribute the SANS data model peak positions where the broad peak position is an indicator of Im3m lattice size and the Lorentz peak position as an indicator of the inverted hexagonal phase (H_{II}) lattice parameter. The observed decrease in lattice parameter i.e. shrinking of the lattice structure in the SANS vs SAXS can be attributed to the presence of buffer (PBS vs water), bulk vs LNP format or differences in intercalation of the F127 stabiliser. In the bulk sample, the F127 is co-lyophilized and therefore evenly distributed. In the LNP case, the distribution is not characterized.

Supplementary Table 6: Statistical analysis of SANS broad peak fit positions as a function of temperature. Data from Supp. Fig. 7B.

Group 1	Group 2	p-value
LNPs at 25 °C	LNPs at 37 °C	0.0489
LNPs at 25 °C	LNPs at 45 °C	0.0000
LNPs at 37 °C	LNPs at 45 °C	0.0001

Supplementary Table 7: SANS data fits as a function of temperature from Fig S7

Temperature (°C)	25	25	25	37	37	37	45	45	45
Scale	1	1	1	1	1	1	1	1	1
Background	0.0034	0.0031	0.0124	-0.0133	0.0114	-0.0220	-0.0060	-0.0026	-0.0062
Background error	0.0151	0.0196	0.0123	0.0088	0.0051	0.0121	0.0045	0.0024	0.0055
A scale	1	1	1	1	1	1	1	1	1
A Porod scale	$1.5492e^{-7}$	$1.7566e^{-7}$	$1.3044e^{-7}$	$1.2805e^{-7}$	$1.2669e^{-7}$	$1.4066e^{-7}$	$1.772e^{-7}$	$1.5006e^{-7}$	$1.6389e^{-7}$
A Porod scale error	$1.3048e^{-7}$	$1.2677e^{-7}$	$1.4687e^{-7}$	$1.9731e^{-8}$	$7.7325e^{-8}$	$2.2281e^{-8}$	$1.8903e^{-8}$	$1.7513e^{-8}$	$1.8578e^{-8}$
A Porod exponential	3.7857	3.7435	3.8142	3.8676	3.8676	3.8464	3.8551	3.8838	3.8660
A Porod exponential error	0.1346	0.1031	0.1065	0.0266	0.0779	0.0282	0.0202	0.0235	0.0195
A Lorentz scale	0.4762	0.4151	0.4195	0.3014	0.2539	0.2369	0.1164	0.0880	0.0935
A Lorentz scale error	0.0519	0.0464	0.0375	0.0123	0.0208	0.0181	0.0082	0.0051	0.0063
A Lorentz length	47.903	44.404	46.036	42.955	50.504	43.572	34.904	36.102	36.874
A Lorentz length error	8.9232	7.7541	6.8246	1.6256	5.8547	2.4803	2.1306	2.6980	2.4281
A Peak position	0.0450	0.0445	0.0459	0.0467	0.0484	0.0473	0.0539	0.0558	0.0561
A Peak position error	0.0040	0.0038	0.0023	0.0006	0.0027	0.0011	0.0020	0.0015	0.0018
A Lorentz exponential	2.5319	2.7604	2.9144	3.1425	2.6061	3.9139	2.8944	5.1301	5.6173
A Lorentz exponential error	0.8841	1.1316	0.6466	0.3377	0.6322	0.6640	0.3252	0.7158	1.2730
B scale	0	0	0	0.0639	0.0383	0.0900	0.1044	0.1117	0.1180
B scale error				0.0109	0.0405	0.0151	0.0156	0.0130	0.0128
B Peak position	-	-	-	0.0976	0.0963	0.0943	0.1022	0.1019	0.1025
B Peak position error				0.0022	0.0079	0.0017	0.0006	0.0006	0.0008
B Peak HWHM	-	-	-	0.0163	0.0093	0.0188	0.0060	0.0069	0.0071
B Peak HWHM error				0.0046	0.0111	0.0042	0.0011	0.0015	0.0021
Im3m Lattice parameter (Å)	139.56	141.17	137.00	134.59	129.77	132.71	116.58	112.69	112.05

Dynamic light scattering data and the changes in the Porod exponent extracted from the SANS data fitting are plotted in figure S8.

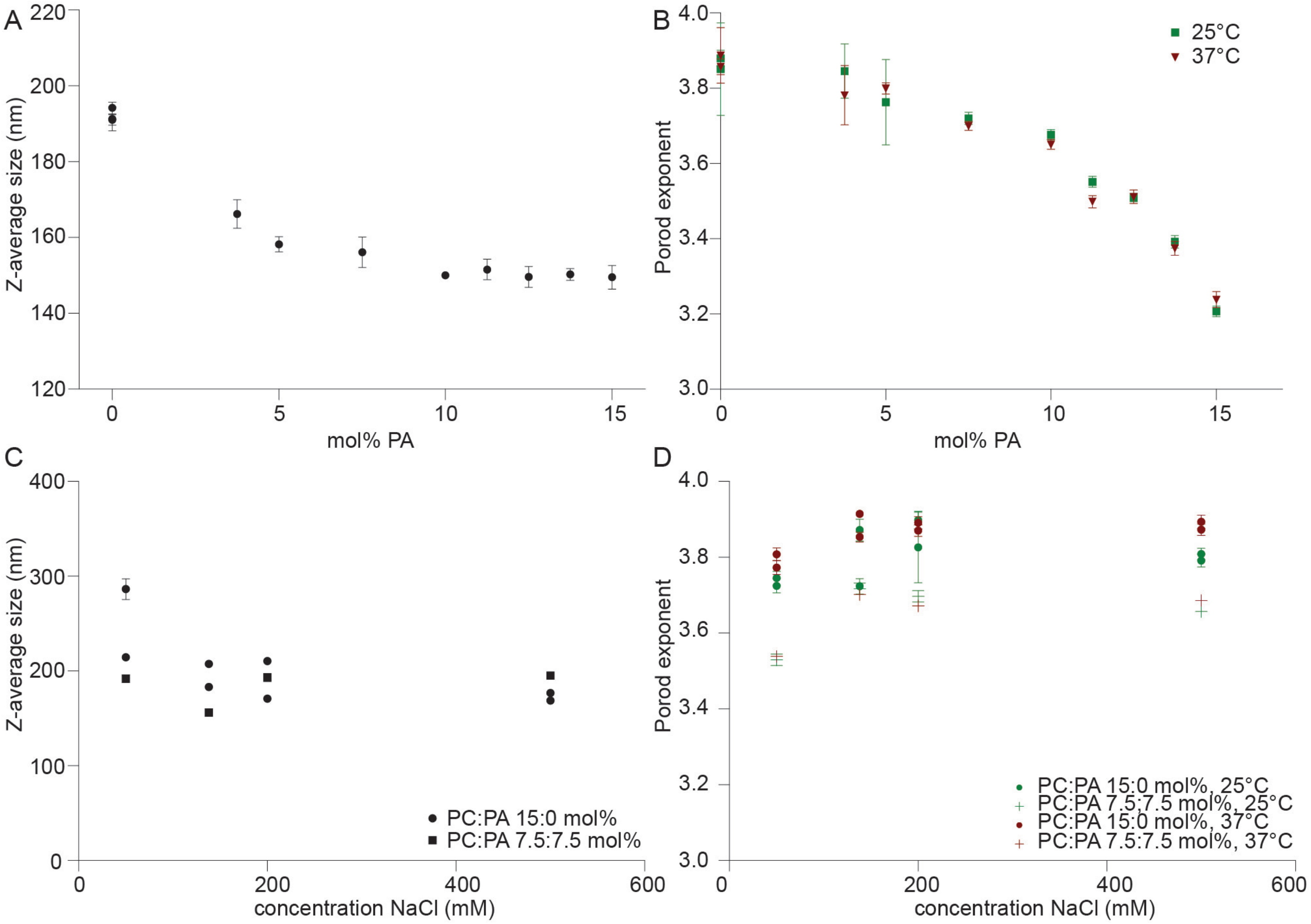

Supplementary Figure 8: SANS and DLS data for LNPs of composition MO:CHOL:DOPC:DOPA 55:30:15-X:X mol% where X = 0, 3.75, 5, 7.5, 10, 11.25, 12.5, 13.75, 15 mol% formulated with 2.5 wt% F127 in 138 mM NaCl (A) DLS data of LNP samples (N = 2 for X = 0, otherwise N = 1, mean and standard deviation of triplicate measurements). (B) Porod exponent as a function of mol% DOPA. (C) DLS data of LNP samples (N = 1, mean and standard deviation of triplicate measurements). (D) Porod exponent as a function of NaCl concentration. For SANS data individual points represent an independent measurement with the fitting error.

Supplementary Table 8: SANS data fits as a function of composition from Fig 3, 25 °C

Temperature	25 °C									
mol% PA	0	0	3.75	5	7.5	10	11.25	12.5	13.75	15
Scale	1	1	1	1	1	1	1	1	1	1
Background	-0.0042	0.0062	-0.0051	0.0058	0.0028	-0.0184	-0.0474	-0.0366	-0.0422	-0.0362
Background error	0.0039	0.0182	0.0112	0.0123	0.0041	0.0053	0.0093	0.0068	0.0127	0.0064
A scale	1	1	1	1	1	1	1	1	1	1
A Porod scale	7.8087e^{-8}	1.1192e^{-7}	1.3530e^{-7}	1.9500e^{-7}	2.9135e^{-7}	3.9544e^{-7}	8.0499e^{-7}	9.8608e^{-7}	1.9971e^{-6}	4.6494e^{-6}
A Porod scale error	8.2649e^{-9}	1.8456e^{-7}	7.9044e^{-8}	1.2736e^{-7}	2.1639e^{-8}	2.7035e^{-8}	5.3494e^{-8}	5.3421e^{-8}	1.5715e^{-7}	2.9871e^{-7}
A Porod exponential	3.8795	3.8509	3.8457	3.7630	3.7199	3.6766	3.5516	3.5099	3.3922	3.2068
A Porod exponential error	0.0213	0.1229	0.0721	0.1135	0.0160	0.0135	0.0148	0.0113	0.0165	0.0141
A Lorentz scale	0.3131	0.4406	0.4057	0.3496	0.3778	0.3947	0.4657	0.4725	0.5161	0.5052
A Lorentz scale error	0.0078	0.0471	0.0290	0.0254	0.0092	0.0102	0.0137	0.0137	0.0220	0.0155
A Lorentz length	42.124	43.003	41.084	44.441	40.809	37.503	37.798	41.489	44.851	50.030
A Lorentz length error	1.0227	6.5371	4.7226	5.2759	0.8550	0.9301	1.2612	1.1407	1.9257	2.0151
A Peak position	0.0429	0.0442	0.0445	0.0450	0.0449	0.0412	0.0384	0.0374	0.0372	0.0354
A Peak position error	0.0004	0.0028	0.0021	0.0026	0.0004	0.0005	0.0005	0.0004	0.0007	0.0005
A Lorentz exponential	2.6788	2.7526	2.7295	2.5251	2.5912	2.0747	1.7283	1.6776	1.5745	1.5131
A Lorentz exponential error	0.1456	0.8933	0.7231	0.5752	0.1291	0.0875	0.0946	0.0723	0.1566	0.0772
B scale	0	0	0	0	0	0	0	0	0	0
B scale error										
B Peak position	-	-	-	-	-	-	-	-	-	-
B Peak position error										
B Peak HWHM	-	-	-	-	-	-	-	-	-	-
B Peak HWHM error										
Lattice parameter (Å)	146.41	142.02	141.28	139.65	139.86	152.63	163.64	167.90	168.91	177.44
Water channel diameter (nm)	5.33	5.06	5.02	4.92	4.93	5.71	6.38	6.64	6.70	7.22

Supplementary Table 9: SANS data fits as a function of composition from Fig 3, 37 °C

Temperature	37 °C									
mol% PA	0	0	3.75	5	7.5	10	11.25	12.5	13.75	15
Scale	1	1	1	1	1	1	1	1	1	1
Background	0.0003	0.0011	0.0017	0.0073	-0.0025	-0.0032	-0.0327	-0.0360	-0.0268	-0.0195
Background error	0.0049	0.0082	0.0062	0.0044	0.0041	0.0076	0.0086	0.0085	0.0082	0.0084
A scale	1	1	1	1	1	1	1	1	1	1
A Porod scale	$1.1212e^{-7}$	$1.1138e^{-7}$	$2.1010e^{-7}$	$1.9107e^{-7}$	$3.6220e^{-7}$	$4.8682e^{-7}$	$1.0687e^{-6}$	$1.0046e^{-6}$	$2.1524e^{-6}$	$3.9478e^{-6}$
A Porod scale error	$1.1850e^{-8}$	$7.1100e^{-8}$	$9.6529e^{-8}$	$1.5653e^{-8}$	$2.0982e^{-8}$	$3.1765e^{-8}$	$8.0452e^{-8}$	$8.5736e^{-8}$	$1.8673e^{-7}$	$3.8410e^{-7}$
A Porod exponential	3.8571	3.8874	3.7815	3.7994	3.6998	3.6507	3.4984	3.5115	3.3748	3.2382
A Porod exponential error	0.0208	0.0738	0.0786	0.0149	0.0122	0.0131	0.0162	0.0181	0.0182	0.0211
A Lorentz scale	0.1767	0.2922	0.2587	0.2155	0.2682	0.2941	0.3915	0.4428	0.4688	0.4710
A Lorentz scale error	0.0087	0.0275	0.0175	0.0065	0.0063	0.0082	0.0145	0.0130	0.0181	0.0198
A Lorentz length	43.550	40.797	39.397	36.829	33.825	34.339	37.189	40.318	42.357	50.231
A Lorentz length error	2.2286	5.7440	3.8647	0.9684	0.7604	1.0985	1.5264	1.5511	2.1106	2.6188
A Peak position	0.0491	0.0475	0.0525	0.0506	0.0480	0.0438	0.0401	0.0381	0.0368	0.0362
A Peak position error	0.0006	0.0029	0.0029	0.0006	0.0006	0.0007	0.0009	0.0006	0.0008	0.0008
A Lorentz exponential	3.2119	2.9420	3.0948	3.5315	2.7376	2.6818	1.8403	1.7044	1.7879	1.6984
A Lorentz exponential error	0.3754	0.6007	0.8923	0.2913	0.1401	0.1362	0.1782	0.1138	0.1284	0.1757
B scale	0.0421	0.0490	0.0119	0.0095	0.0095	0.0035	0	0	0	0
B scale error	0.0209	0.0388	0.0500	0.0049	0.0062	0.0076				
B Peak position	0.0972	0.0978	0.0974	0.0979	0.1196	0.0917	-	-	-	-
B Peak position error	0.0014	0.0069	0.0098	0.0097	0.0116	0.0101				
B Peak HWHM	0.0076	0.0101	0.0032	0.0285	0.0035	1.5402	-	-	-	-
B Peak HWHM error	0.0018	0.0129	0.0075	0.0323	0.0730	5051.8				
Lattice parameter (Å)	127.86	132.36	119.68	124.24	130.96	143.48	156.63	164.83	170.81	173.36
Water channel diameter (nm)	4.20	4.47	3.70	3.98	4.39	5.15	5.95	6.45	6.82	6.97

Supplementary Table 10: SANS data fits as a function of salt concentration from Fig 3, 25 °C

Temperature	25 °C											
mol% PA	0	0	0	0	0	0	0	0	7.5	7.5	7.5	7.5
NaCl conc (mM)	50	138	200	500	50	138	200	500	50	138	200	500
Scale	1	1	1	1	1	1	1	1	1	1	1	1
Background	0.0224	0.0221	0.0051	0.0115	0.0188	-0.0001	0.0031	0.0039	0.0223	0.0023	-0.0012	0.0010
Background error	0.0026	0.0032	0.0136	0.0027	0.0029	0.0053	0.0032	0.0031	0.0031	0.0035	0.0026	0.0032
A scale	1	1	1	1	1	1	1	1	1	1	1	1
A Porod scale	$1.8557e^{-7}$	$1.8491e^{-7}$	$1.2664e^{-7}$	$1.4466e^{-7}$	$1.5340e^{-7}$	$8.1598e^{-8}$	$7.4400e^{-8}$	$1.3680e^{-7}$	$8.0002e^{-7}$	$2.9357e^{-7}$	$3.1497e^{-7}$	$4.3422e^{-7}$
A Porod scale error	$1.5824e^{-8}$	$1.9049e^{-8}$	$9.6487e^{-8}$	$1.0873e^{-8}$	$1.3792e^{-8}$	$1.2715e^{-8}$	$9.3008e^{-9}$	$1.0935e^{-8}$	$5.6920e^{-8}$	$1.9998e^{-8}$	$2.2060e^{-8}$	$2.3532e^{-8}$
A Porod exponential	3.7245	3.7235	3.8259	3.8085	3.7454	3.8718	3.8979	3.7913	3.5297	3.7175	3.6972	3.6571
A Porod exponential error	0.0181	0.0206	0.0927	0.0148	0.0179	0.0285	0.0233	0.0170	0.0151	0.0150	0.0148	0.0115
A Lorentz scale	0.4483	0.4470	0.4402	0.3795	0.3265	0.3085	0.3141	0.2854	0.3423	0.3797	0.3546	0.2767
A Lorentz scale error	0.0060	0.0069	0.0437	0.0060	0.0060	0.0085	0.0070	0.0060	0.0068	0.0076	0.0066	0.0058
A Lorentz length	44.692	44.655	45.543	46.599	42.399	43.014	45.225	47.258	27.457	41.213	45.519	44.769
A Lorentz length error	0.6500	0.7574	4.9558	0.8083	0.8578	1.2313	0.9280	1.0298	2.1136	0.8628	0.8748	0.8711
A Peak position	0.0445	0.0446	0.0456	0.0484	0.0431	0.0430	0.0451	0.0482	0.0318	0.0448	0.0474	0.0537
A Peak position error	0.0003	0.0003	0.0016	0.0003	0.0004	0.0005	0.0004	0.0003	0.0031	0.0004	0.0003	0.0003
A Lorentz exponential	3.1073	3.1065	2.9516	3.0659	2.9578	2.8014	2.9462	2.7058	3.6780	2.5507	2.8273	2.7820
A Lorentz exponential error	0.1107	0.1329	0.7446	0.1171	0.1512	0.1818	0.1770	0.1527	0.3302	0.1180	0.1256	0.1406
B scale	0	0	0	0	0	0	0	0	0	0	0	0
B scale error												
B Peak position	-	-	-	-	-	-	-	-	-	-	-	-
B Peak position error												
B Peak HWHM	-	-	-	-	-	-	-	-	-	-	-	-
B Peak HWHM error												
Lattice parameter (Å)	141.33	140.92	137.64	129.72	145.63	146.26	139.37	130.38	197.82	140.10	132.68	117.05
Water channel diameter (nm)	5.02	5.00	4.80	4.31	5.28	5.32	4.90	4.35	8.47	4.95	4.49	3.54

Supplementary Table 11: SANS data fits as a function of salt concentration from Fig 3, 37 °C

Temperature	37 °C											
mol% PA	0	0	0	0	0	0	0	0	7.5	7.5	7.5	7.5
NaCl conc (mM)	50	138	200	500	50	138	200	500	50	138	200	500
Scale	1	1	1	1	1	1	1	1	1	1	1	1
Background	0.0047	-0.0116	-0.0060	-0.0130	0.0037	-0.0030	-0.0060	-0.0105	0.0103	0.0025	-0.0102	0.0035
Background error	0.0033	0.0081	0.0110	0.0049	0.0060	$2.7241e^{-9}$	0.0050	0.0062	0.0034	0.0035	0.0038	0.0036
A scale	1	1	1	1	1	1	1	1	1	1	1	1
A Porod scale	$1.8597e^{-7}$	$1.3834e^{-7}$	$1.2888e^{-7}$	$1.1661e^{-7}$	$1.3916e^{-7}$	$8.6258e^{-8}$	$9.5974e^{-8}$	$1.1008e^{-7}$	$8.3226e^{-7}$	$3.5954e^{-7}$	$4.0411e^{-7}$	$4.0576e^{-7}$
A Porod scale error	$1.6471e^{-8}$	$8.2414e^{-9}$	$9.3188e^{-9}$	$1.1299e^{-8}$	$9.3647e^{-9}$	$7.1175e^{-14}$	$7.8208e^{-9}$	$8.0691e^{-9}$	$4.8157e^{-8}$	$2.1817e^{-8}$	$2.2533e^{-8}$	$1.9452e^{-8}$
A Porod exponential	3.7730	3.8534	3.8705	3.8940	3.8078	3.9149	3.8912	3.8726	3.5391	3.7017	3.6719	3.6864
A Porod exponential error	0.0182	0.0126	0.0153	0.0174	0.0169	$3.3401e^{-6}$	0.0162	0.0150	0.0119	0.0120	0.0112	0.0102
A Lorentz scale	0.2901	0.2894	0.2718	0.2366	0.1780	0.1866	0.1775	0.1645	0.2622	0.2652	0.2439	0.1897
A Lorentz scale error	0.0054	0.0081	0.0114	0.0058	0.0080	$1.5065e^{-7}$	0.0070	0.0060	0.0063	0.0060	0.0067	0.0058
A Lorentz length	43.917	45.182	47.055	43.319	39.378	42.317	43.302	45.462	24.235	34.548	38.864	41.616
A Lorentz length error	0.8964	1.0167	1.7156	2.0198	2.0341	$3.827e^{-5}$	2.2778	2.1677	1.9863	0.8326	0.8467	0.9353
A Peak position	0.0477	0.0468	0.0480	0.0500	0.0484	0.0472	0.0489	0.0500	0.0334	0.0481	0.0521	0.0588
A Peak position error	0.0004	0.0004	0.0005	0.0006	0.0007	$3.8368e^{-8}$	0.0006	0.0007	0.0043	0.0005	0.0004	0.0004
A Lorentz exponential	3.3118	3.6171	3.3410	2.7870	3.2249	2.9335	2.9685	3.6668	3.8468	2.7773	2.7257	2.8905
A Lorentz exponential error	0.2116	0.2927	0.4086	0.3881	0.2292	$2.6325e^{-6}$	0.4763	0.4136	0.4332	0.1548	0.1967	0.2239
B scale	0.0539	0.0704	0.0593	0.0636	0.0432	0.0282	0.0417	0.0559	0	0	0	0
B scale error	0.0054	0.0074	0.0140	0.0097	0.0098	$2.3113e^{-8}$	0.0073	0.0088				
B Peak position	0.0957	0.0950	0.0951	0.0966	0.0981	0.0974	0.0951	0.0939	-	-	-	-
B Peak position error	0.0012	0.0019	0.0026	0.0018	0.0011	$3.9544e^{-8}$	0.0015	0.0025				
B Peak HWHM	0.0113	0.0203	0.0151	0.0130	0.0089	0.0116	0.0097	0.0191	-	-	-	-
B Peak HWHM error	0.0023	0.0034	0.0049	0.0028	0.0022	$8.8056e^{-9}$	0.0041	0.0052				
Lattice parameter (Å)	131.66	134.21	130.81	125.71	129.70	133.06	128.44	125.56	187.88	130.56	120.52	106.90
Water channel diameter (nm)	4.43	4.59	4.38	4.07	4.31	4.52	4.24	4.06	7.86	4.36	3.75	2.92

Supplementary Table 12: SANS data fitting and lattice parameter calculations for PLD incubations

	Active PLD			No PLD			Inactive PLD		
Scale	1	1	1	1	1	1	1	1	1
Background	-0.0044	0.0067	-0.0076	0.0256	0.0179	0.0243	0.0214	0.0191	0.0255
Background error	0.0090	0.0098	0.0100	0.0026	0.0030	0.0028	0.0059	0.0047	0.0040
A scale	1	1	1	1	1	1	1	1	1
A Porod scale	2.1559e^{-7}	1.6861e^{-7}	1.9858e^{-7}	9.2581e^{-8}	8.3461e^{-8}	1.0573e^{-7}	2.4340e^{-7}	2.2354e^{-7}	2.2986e^{-7}
A Porod scale error	1.4905e^{-8}	2.2643e^{-8}	1.7207e^{-8}	7.4673e^{-9}	7.1669e^{-9}	9.6032e^{-9}	1.8423e^{-8}	2.3259e^{-8}	2.2352e^{-8}
A Porod exponential	3.8372	3.8800	3.8531	3.8613	3.8753	3.8303	3.7183	3.7226	3.7255
A Porod exponential error	0.0182	0.0229	0.0216	0.0163	0.0168	0.0194	0.0152	0.0225	0.0179
A Lorentz scale	0.1550	0.0152	0.1481	0.4446	0.4223	0.4341	0.3442	0.3127	0.3089
A Lorentz scale error	0.0130	0.0171	0.0178	0.0062	0.0067	0.0061	0.0107	0.0085	0.0084
A Lorentz length	46.650	50.128	42.067	46.869	45.504	45.905	41.199	38.748	41.115
A Lorentz length error	2.3327	4.9485	5.6962	0.6891	0.8213	0.7442	1.1572	1.3211	1.4176
A Peak position	0.0525	0.0522	0.0545	0.0449	0.0443	0.0446	0.0449	0.0434	0.0441
A Peak position error	0.0010	0.0016	0.0012	0.0003	0.0003	0.0003	0.0004	0.0007	0.0005
A Lorentz exponential	2.5256	2.4018	2.7931	3.0821	2.8692	3.0728	2.3429	2.5201	2.5956
A Lorentz exponential error	0.1973	1.2807	0.4301	0.1153	0.1154	0.1136	0.1467	0.2090	0.1616
B scale	0.0622	0.0612	0.0719	0	0	0	0	0	0
B scale error	0.0093	0.0126	0.0125						
B Peak position	0.0993	0.0961	0.1060	-	-	-	-	-	-
B Peak position error	0.0075	0.0091	0.0063						
B Peak HWHM	0.0489	0.0533	0.0504	-	-	-	-	-	-
B Peak HWHM error	0.0312	0.0108	0.0180						
Lattice parameter (Å)	119.57	120.34	115.19	139.87	141.77	141.01	139.97	144.77	142.62
Water channel diameter (nm)	3.69	3.74	3.43	4.93	5.05	5.00	4.94	5.23	5.10

In this analysis, the focus is specifically on the broad peak position, however it should be noted that the SANS characterization also facilitates deeper analytical analysis. The broad peak function is a Lorentzian peak plus a power law decay and therefore the SANS analysis provides information on the power law scale factor (Porod scale), power law exponent (Porod exponent), Lorentzian peak scale factor (Lorentz scale), Lorentzian screening length (Lorentz length i.e. peak width) and the exponent of the Lorentz peak (Lorentz exponent). The extracted parameters for all data fits performed have been included in the preceeding data tables. Of particular note, as a function of temperature, the Lorentz length and scale showed a decreasing trend with increasing temperature. This indicates increasing disorder in the LNP internal morphology and a reduction in the peak intensity respectively. Increasing the temperature of monoolein (25 - 45 °C) in excess water, leads to increased lipid chain conformational disorder and a decrease in the size of the lattice structure.[2] This behaviour is mirrored in the SANS data via the broad peak position (structure size) and Lorentz length (ordering of lattice repeat units). With increasing temperature, both the SAXS and SANS data indicated the formation of a coexisting inverted hexagonal H_{II} phase leading to a reduction of the proportion of bicontinuous cubic (Im3m) phase present in the LNP sample. This validates the corresponding reduction observed in the Lorentz scale with increasing temperature. As a function of increasing DOPA content in the LNPs, trends in the Porod scale and exponent were particularly notable. This may indicate changes in gross morphology or large scale structure / contrast changes. Both parameters showed a decreasing trend with increasing DOPA content. It should be noted that for SANS static measurements, LNPs were formulated with different DOPC:DOPA ratios and the system is therefore assumed to be under equilibrium. DLS measurements showed that from 0 to 7.5 mol% DOPA, Z-Ave size decreased from approximately 190 nm to 158 nm. From 7.5 mol% to 15 mol%, the Z-Ave size only decreased from 156 to 150 nm (fig. S8). Therefore the observed trends in the Porod scale and exponent are attributed to LNP composition and not size variations. Specifically they indicate that increasing the amount of anionic charge in the LNP formulation, may reduce the surface roughness of the LNP, where a Porod exponent of 4 is indicative of a sphere or smooth surface and reduction an increased roughness. The Porod scale factor is more complex and may indicate an increase in the variability of the population within the sample suggesting a volume fraction change or a change in contrast e.g. due to the presence of sub populations. In the case of electrostatic tuning via NaCl concentration, aside from the peak position, no other trends were noted for the 15 mol% LNPs whilst for the 7.5 mol% LNP, the Porod exponent decreased and the Porod scale increased with decreasing NaCl concentration. The scattering intensities in the low q regime (Guinier region) indicate that a small number of aggregates may be present, however all samples measured via DLS exhibited a PDI below 0.2. There is also little variation in this scattering as a function of lipid composition and salt concentration, agreeing with the DLS data that whilst structural variations are observed in the LNPs, there is little change in the overall LNP size. For the static measurements, LNPs were formulated at the stated compositions and therefore the assumption is that all LNPs are of similar composition and that the lipids are evenly distributed across the particles. It has not yet been demonstrated what the distribution of the constituent lipids within this LNP system is, however, it is likely to be predominantly uniform throughout the LNP population.

Supplementary Table 13: Statistical analysis of LNP formulations DLS measurements of Z-Ave size for LNPs with and without PLD. Data from Fig. 4b.

Formulation	Group 1	Group 2	p-value
	No PLD	Active PLD	0.0027
MO55 PA00 PC15 Ch30	No PLD	Inactive PLD	0.0074
	Active PLD	Inactive PLD	0.5585

Supplementary Table 14: Statistical analysis of SANS fitting parameters for LNPs with and without PLD. Data from Fig. 4c and d.

Parameter	Group 1	Group 2	p-value
	No PLD	Active PLD	0.0000
Peak position	No PLD	Inactive PLD	0.7794
	Active PLD	Inactive PLD	0.0000
	No PLD	Active PLD	0.0004
Lorentz scale	No PLD	Inactive PLD	0.0622
	Active PLD	Inactive PLD	0.0034

1.5 Comparison of SAXS / SANS bulk and LNP analysis

The SANS observations are in agreement with the bulk data trends and demonstrates that SANS data models can be applied to internal morphology characterization of LNPs.

Supplementary Table 15: Comparison of SAXS and SANS fit parameters from experimental data

	SAXS (Å)				SANS (Å)			
	Im3m		H_{II}		Broad peak		Lorentz peak	
T (°C)	Mean	Fit error	Mean	Fit error	Mean	St. Dev.	Mean	St. Dev.
25	191.788	0.2223	90.136	0.1724	139.245	2.1021	-	-
37	150.642	0.2779	73.275	0.0364	132.359	2.4268	75.526	1.2853
45	-	-	-	-	113.774	2.4474	71.007	0.2165

However, there are notable differences in the observed water channel diameters between the bulk and LNP samples. The bulk data was measured in MQ water and the LNPs in PBS and therefore electrostatic screening of the anionic charge as a result of the PBS salt content is expected. This results in a reduction of the water channel diameter swelling at the equivalent mol% of DOPA. This is also likely to shift the Im3m - lamellar phase transition observed in the bulk sample to higher mol% of DOPA i.e. a higher amount of DOPA is needed to produced the same water channel diameter swelling in PBS

compared to water. The SANS data shows no evidence of an Im3m - lamellar phase transition, but if a similar swelling in the water channel diameter was obtained we would expect to see this phase change reflected in the SANS data. Incorporation of the F127 into the bulk phase also assumes a complete intercalation of the F127 stabiliser, whereas in the LNP if the F127 is primarily surface based, this would reduce the water channel diameter.[3] Furthermore, conversion of the broad peak position to a lattice parameter is an approximation. In the SAXS data, the peaks within a single phase are individually assigned and a lattice parameter calculated whilst for the SANS data the individual peaks are not resolved and therefore a single position represents an average of the individual peaks. Nevertheless, the data represents use of SANS for the determination of LNP internal morphology as a function of temperature and composition.

1.6 PLD mediated SAXS analysis

To validate if PLD activity could induce similar morphological changes in LNPs via active conversion of DOPC to DOPA, SAXS of LNPs incubated with PLD was performed. LNPs formulated in PBS were highly prone to radiation damage and it was therefore not possible to track the structural evolution continuously. Instead, dispersions and PLD were mixed and co incubated in a capillary at 37 °C to maximise PLD activity and SAXS data obtained at approximately 15 and 30 mins post PLD addition. Capillaries with either inactive PLD or no PLD were used as a control. At 15 and 30 mins the sample with no PLD exhibited a predominantly inverted hexagonal phase morphology (H_{II}, lattice parameters 73.6 ± 0.1 Å and 73.5 ± 0.1 Å respectively). A weak coexistence with an Im3m phase (lattice parameter 180.4 ± 5.1 Å) was also observed. The H_{II} lattice parameter agrees well with the lattice parameter observed at 37 °C in bulk and the Im3m lattice parameter is 30 Å larger. Some heterogeneity of lattice parameter has been observed in LNP samples with defined phase morphologies - it is also as yet unknown what the level of intercalation of the F127 is in each LNP population, which may explain the differences observed. In the samples incubated with inactive PLD an H_{II} lattice parameter of 75.3 ± 0.5 Å and 75.7 ± 0.5 Å was observed respectively. The PLD is supplied in glycerol so it is possible that via the addition of inactive PLD, the H_{II} lattice parameter is increased as a result. In the capillaries incubated with active PLD, at 15 minutes an H_{II} lattice parameter of 74.9 ± 0.4 Å was observed and at 30 mins an H_{II} lattice parameter of 70.1 ± 0.3. A small Im3m component is visible in coexistence but there is not significant resolution to fit the Im3m phase. Additionally broadening of the H_{II} phase was observed in comparison to the controls implying an increase in the disorder of the LNP internal structure. This increase in disorder in the internal LNP structure and potential formation of the H_{II} phase was supported by Cryo TEM imaging (Figure S10). This indicates that the influence of PLD activity on LNP morphology may differ from bottom up formulation of the expected lipid compositions.

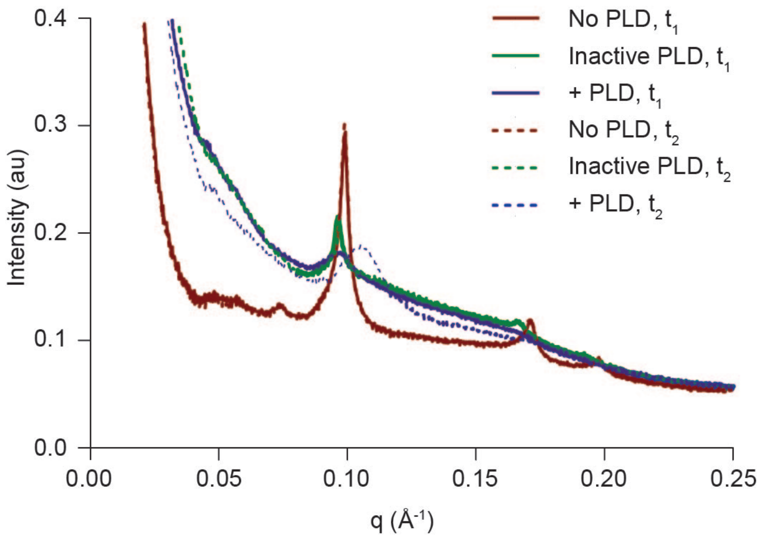

Supplementary Figure 9: SAXS diffraction data from an LNP sample of MO:CHOL:DOPC 55:30:15 mol% + 2.5 wt% F127 hydrated in 95 wt% PBS and incubated with PLD at 37°C, where t_1 = 15 mins, t_2 = 30 mins and no enzyme (No PLD), inactive enzyme (Inactive PLD), and active enzyme (+ PLD).

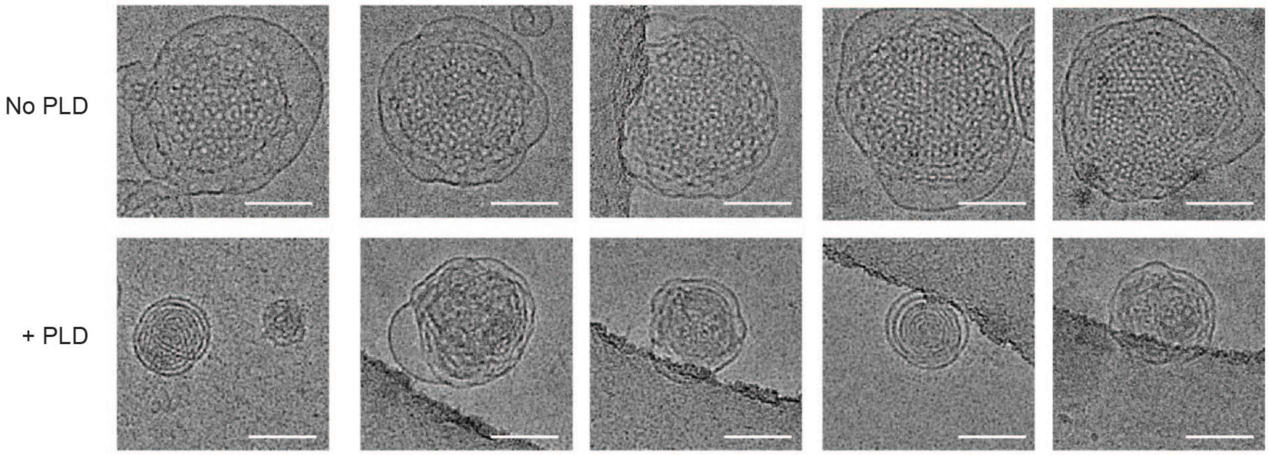

Supplementary Figure 10: Representative Cryo TEM data of MO:CHOL:DOPC 55:30:15 mol% LNPs imaged prior to PLD addition and 80 minutes after PLD addition. The scale bar is 100 nm in all images.

1.7 Additional film and vesicle SPARTA® data

Raman spectral data was obtained to characterize lipid films for 100% DOPC and 100% DOPA prior to vesicle formation via film hydration and extrusion (Fig. S11A). Additionally example spectra from a DOPC lipid film was compared to an average spectrum obtained from trapping 200 nm diameter extruded 100% DOPC vesicles (Fig. S11B).

It is worth noting that the vesicle formulations were less efficiently trapped compared to the LNPs and yielded overall weaker signals. This may be due to the reduced total number of lipids per particle, existing in the ideally-unilamellar vesicle, versus the bicontinuous cubic phase of the LNP. This reduced lipid content may provide a lower overall change in the refractive index and polarizability of the nanoparticle, which may impact the efficacy of the SPARTA® optical trap, sup-

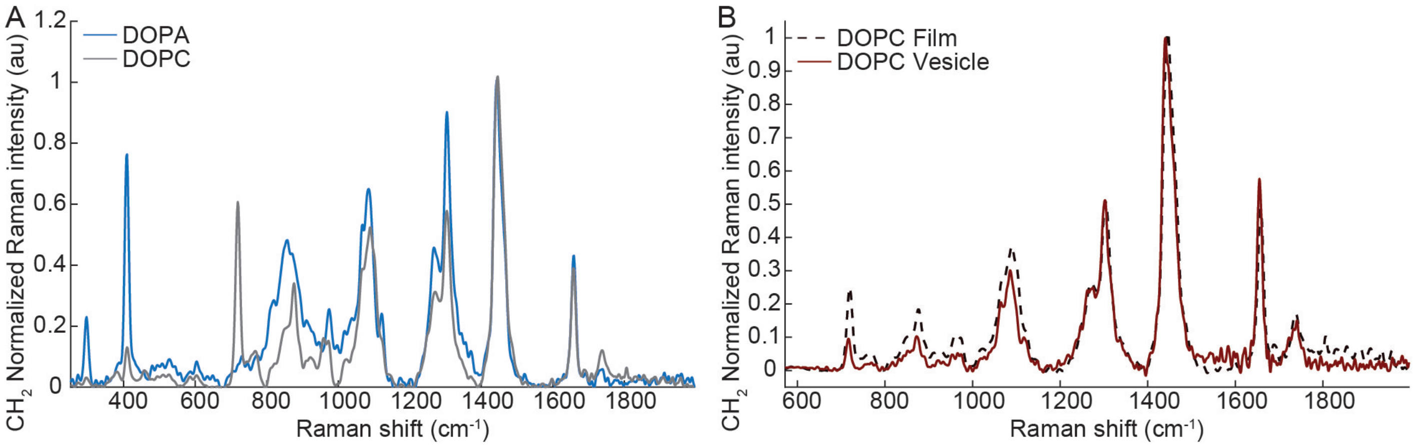

Supplementary Figure 11: Lipid film and vesicle characterization. Measured lipid spectra for (A) DOPA & DOPC films, and (B) DOPC film and SPARTA® trapped extruded vesicles. The acquired spectra indicate composition differences between lipid films and correspondence between lipid film and trapped DOPC vesicles.

ported by the increased trapping efficiency of 200 nm vesicles relative to 100 nm vesicles in Fig. 5d.
Comparison of extended incubation of lipid vesicle formulations with high concentration phospholipase D (Fig. S12) indicates that, independent of cholesterol incorporation into the formulation, there is no significant detectable activity of the PLD on vesicle structures. In fact, even extending the incubation time did not reveal consistent and detectable changes in these vesicle formulations within 3 hours after incubation. This is directly opposed to the LNP time course that approached full conversion of the PC substrate in under 15 minutes for the same concentration of enzyme and DOPC mass.

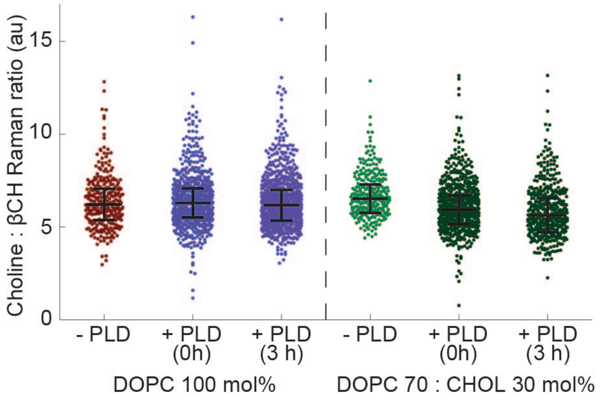

Supplementary Figure 12: Reduced phospholipase D activity on extruded vesicles formulated with and without cholesterol. (Left) Neat DOPC vesicles and those incubated with 280 U/mL PLD for 0 or ≥3 hours prior to initializing SPARTA® trapping time course and (right) DOPC:CHOL mol% 70:30 neat and incubated with 280 U/mL PLD for 0 or ≥3 hours prior to SPARTA enzyme time course evaluation (n ≥ 290). Compared to LNPs and control formulation vesicles with DOPC:DOPA ratios, no change in the composition of individually trapped vesicles is detected via SPARTA over this time course with the matched enzyme concentration.

Supplementary Table 16: Statistical analysis of control vesicle formulations with SPARTA. Data from Fig. 5b

Group 1	Group 2	p-value
DOPA 0 DOPC 100	DOPA 10 DOPC 90	0.0000
DOPA 0 DOPC 100	DOPA 20 DOPC 80	0.0000
DOPA 0 DOPC 100	DOPA 30 DOPC 70	0.0000
DOPA 0 DOPC 100	DOPA 40 DOPC 60	0.0000
DOPA 0 DOPC 100	DOPA 50 DOPC 50	0.0000
DOPA 10 DOPC 90	DOPA 20 DOPC 80	0.3050
DOPA 10 DOPC 90	DOPA 30 DOPC 70	0.0120
DOPA 10 DOPC 90	DOPA 40 DOPC 60	0.0001
DOPA 10 DOPC 90	DOPA 50 DOPC 50	0.0032
DOPA 20 DOPC 80	DOPA 30 DOPC 70	0.9504
DOPA 20 DOPC 80	DOPA 40 DOPC 60	0.9377
DOPA 20 DOPC 80	DOPA 50 DOPC 50	0.6919
DOPA 30 DOPC 70	DOPA 40 DOPC 60	1.0000
DOPA 30 DOPC 70	DOPA 50 DOPC 50	0.9860
DOPA 40 DOPC 60	DOPA 50 DOPC 50	0.9486

Supplementary Table 17: Statistical analysis of vesicle formulations of differing sizes with and without PLD via SPARTA. Data from Fig. 5d.

Formulation	Group 1	Group 2	p-value
DOPC 100% 100nm	No PLD	Active PLD	0.8906
DOPC 100% 200nm	No PLD	Active PLD	0.0097

Supplementary Table 18: Statistical analysis of vesicle formulations with SPARTA with extended PLD incubation. Data from Supp. Fig. 12.

Formulation	Group 1	Group 2	p-value
DOPC 100% 200nm	No PLD	Active PLD, no incubation	0.7134
	No PLD	Active PLD, 3Hr incubation	0.9406
	Active PLD, no incubation	Active PLD, 3Hr incubation	0.2817
DOPC 70% Chol 30% 200nm	No PLD	Active PLD, no incubation	0.0000
	No PLD	Active PLD, 3Hr incubation	0.0000
	Active PLD, no incubation	Active PLD, 3Hr incubation	0.0005

1.8 Additional fluorescence and DLS data

This section details statistical analysis of the fluorescence data and additional fluorescence cascade assay data for LNPs and vesicles at long timepoints (6 days), assay calibration as a function of DOPC concentration and PLD activity calculations for DOPC concentrations of 16.7μg/mL or 83.4μg/mL.

Supplementary Table 19: Statistical analysis of PLD activity on LNPs via fluorescence cascade assay as a function of PLD concentration. Data from Fig. 6c.

Formulation	Group 1	Group 2	p-value
	11 U/mL PLD	22 U/mL PLD	0.1838
MO55 PA00 PC15 Ch30	11 U/mL PLD	44 U/mL PLD	0.0698
	22 U/mL PLD	44 U/mL PLD	0.7379

Supplementary Table 20: Statistical analysis of PLD activity on LNPs and vesicles via fluorescence cascade assay as a function of PLD concentration. Data from Supp. Fig. 13C.

Group 1	Group 2	p-value
LNP, 44 U/mL PLD	LNP, 22 U/mL PLD	0.4712
LNP, 44 U/mL PLD	LNP, 11 U/mL PLD	0.0076
LNP, 44 U/mL PLD	Ves 100nm, 44 U/mL PLD	0.0001
LNP, 44 U/mL PLD	Ves 200nm, 44 U/mL PLD	0.0001
LNP, 22 U/mL PLD	LNP, 11 U/mL PLD	0.1037
LNP, 22 U/mL PLD	Ves 100nm, 44 U/mL PLD	0.0010
LNP, 22 U/mL PLD	Ves 200nm, 44 U/mL PLD	0.0011
LNP, 11 U/mL PLD	Ves 100nm, 44 U/mL PLD	0.0638
LNP, 11 U/mL PLD	Ves 200nm, 44 U/mL PLD	0.0655
Ves 100nm, 44 U/mL PLD	Ves 200nm, 44 U/mL PLD	1.000

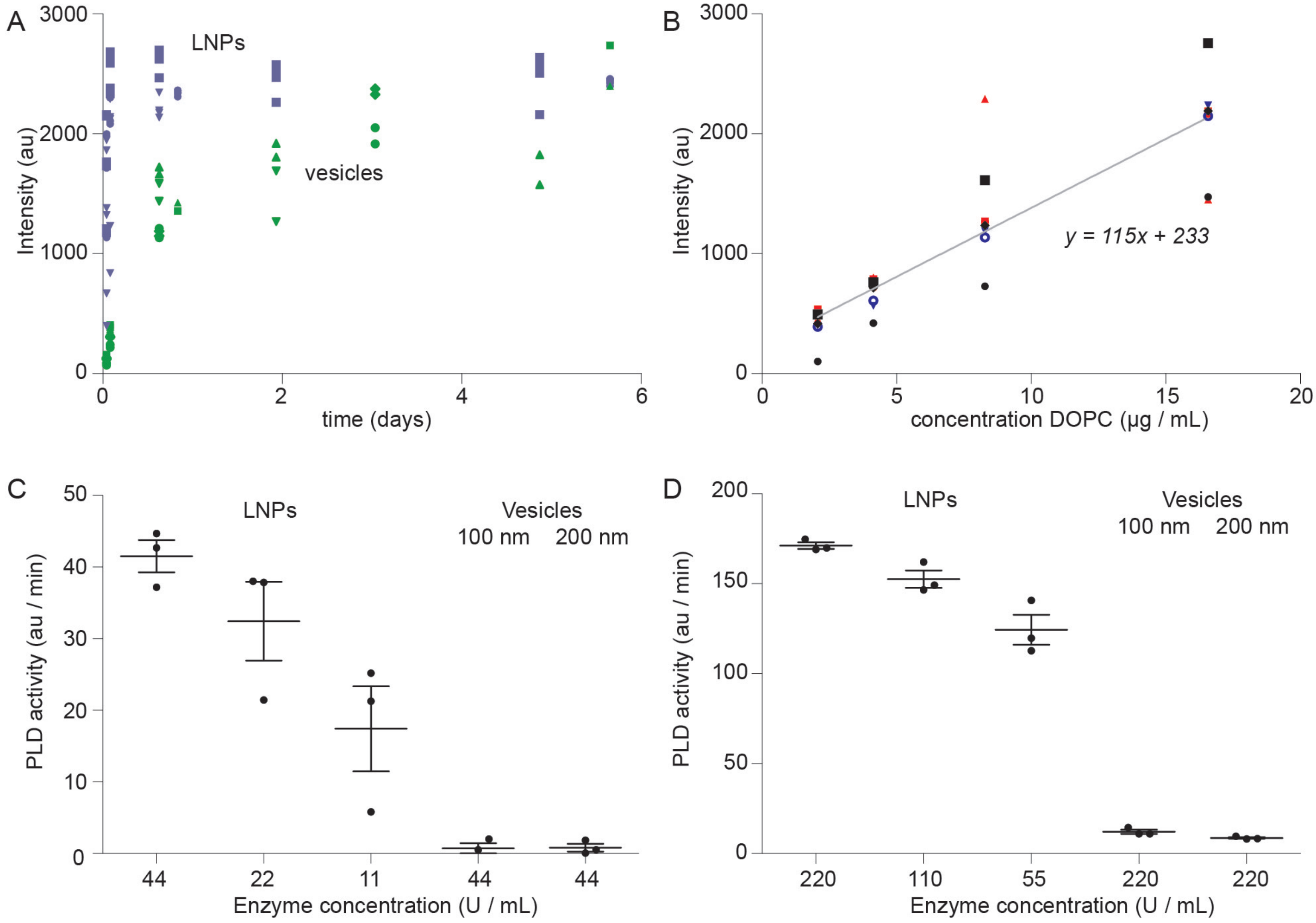

Supplementary Figure 13: A) Pooled data (N = 3) of long timepoints of DOPC = 16.7μg/mL. Wells that showed a downward trend in fluorescence have been excluded due to evaporation. Each shape represents an individual experiment and each data point represents a single measurement. LNP data is represented in purple and vesicle data in green. (B) Calibration curve for LNPs and vesicles using 50× enzyme (vesicles), 10× enzyme (LNPs) to measure endpoints (each datapoint represents a measurement, colors represent independent samples). Data is pooled from 3 independent samples each of LNPs and vesicles. (C,D) Rates (mean ± standard deviation, N = 3) obtained from linear fitting of fluorescence data using either (C) DOPC = 16.7μg/mL or (D) DOPC = 83.4μg/mL. In both cases the lipid : enzyme ratio is identical. Linear fits were made to 5 datapoints around the midpoint for the maximum fluorescence. The maximum fluorescence is defined as in each well for LNPs and the average of all the LNP maxima for the vesicles.

Supplementary Table 21: Statistical analysis of PLD activity on LNPs and vesicles via fluorescence cascade assay as a function of PLD concentration. Data from Fig. 6d and Supp. Fig. 13D.

Group 1	Group 2	p-value
LNP, 220 U/mL PLD	LNP, 110 U/mL PLD	0.0802
LNP, 220 U/mL PLD	LNP, 55 U/mL PLD	0.0002
LNP, 220 U/mL PLD	Ves 100nm, 220 U/mL PLD	0.0000
LNP, 220 U/mL PLD	Ves 200nm, 220 U/mL PLD	0.0000
LNP, 110 U/mL PLD	LNP, 55 U/mL PLD	0.0078
LNP, 110 U/mL PLD	Ves 100nm, 220 U/mL PLD	0.0000
LNP, 110 U/mL PLD	Ves 200nm, 220 U/mL PLD	0.0000
LNP, 55 U/mL PLD	Ves 100nm, 220 U/mL PLD	0.0000
LNP, 55 U/mL PLD	Ves 200nm, 220 U/mL PLD	0.0000
Ves 100nm, 220 U/mL PLD	Ves 200nm, 220 U/mL PLD	0.9799

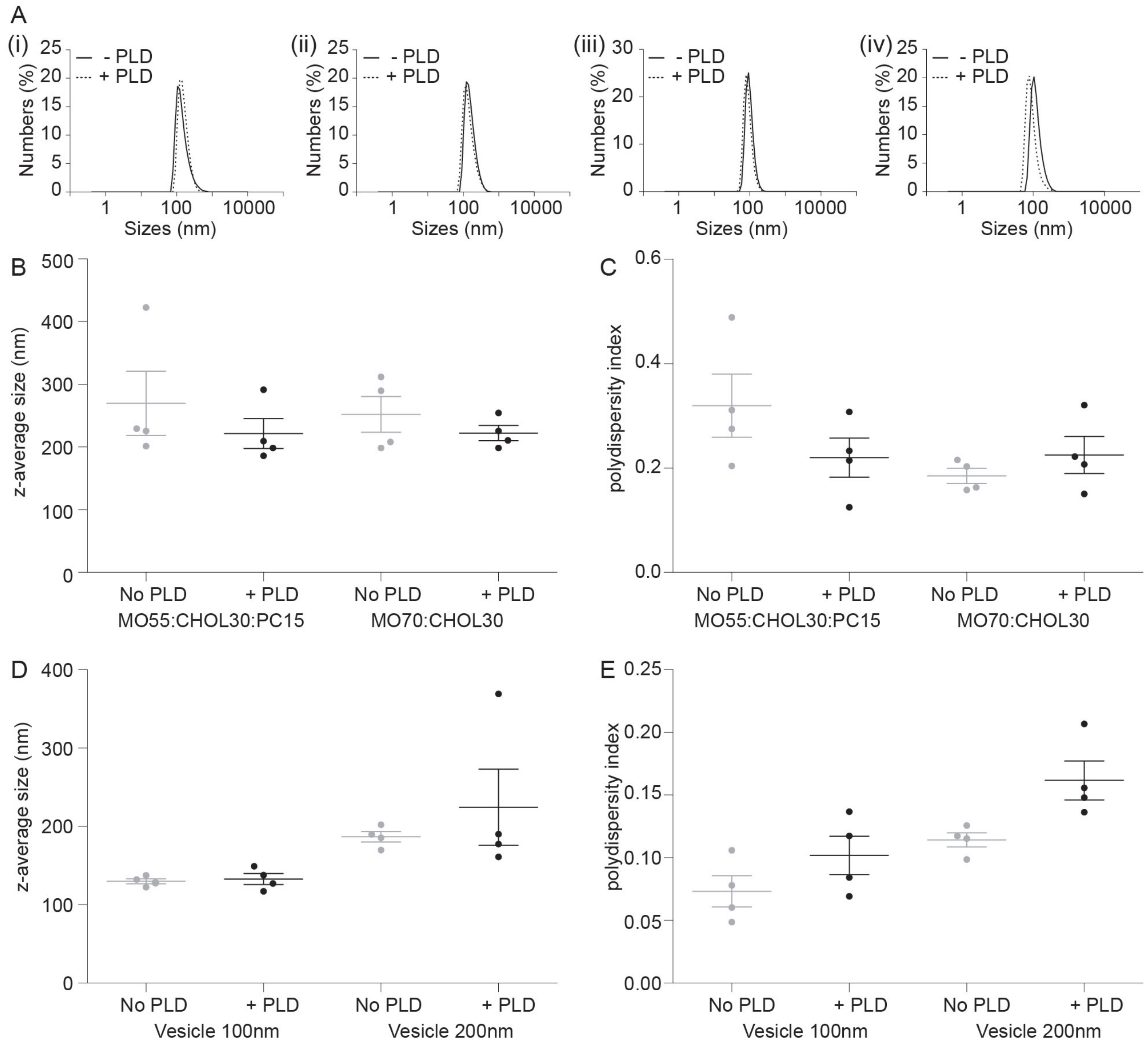

Supplementary Figure 14: (A) Representative DLS traces for the numbers (%) data for LNPs and vesicles + PLD (dotted line) and - PLD (solid line) where, (i) LNPs containing MO:CHOL:DOPC 55:30:15 mol%, (ii) LNPs containing MO:CHOL 70:30 mol%, (iii) DOPC vesicles (100 nm), (iv) DOPC vesicles (200 nm), (B) Z-Ave size (diameter, nm) for LNPs + and - PLD (N = 4, mean ± standard error), (C) Polydispersity index for LNPs and vesicles + and - PLD (N = 4, mean ± standard error), (D) Z-Ave size (diameter, nm) for vesicles + and - PLD (N = 4, mean ± standard error), (E) Polydispersity index for vesicles + and - PLD (N = 4, mean ± standard error).

Supplementary Table 22: Statistical analysis of PLD activity on LNPs and vesicles via DLS analysis of Z-average size. Data from Supp. Fig. 14B and D.

Formulation	Group 1	Group 2	p-value
LNP	MO55 PA00 PC15 Ch30, No PLD	MO55 PA00 PC15 Ch30, Active PLD	0.6101
	MO55 PA00 PC15 Ch30, No PLD	MO70 PA00 PC00 Ch30, No PLD	0.9961
	MO55 PA00 PC15 Ch30, No PLD	MO70 PA00 PC00 Ch30, Active PLD	0.9219
	MO55 PA00 PC15 Ch30, Active PLD	MO70 PA00 PC00 Ch30, No PLD	0.7475
	MO55 PA00 PC15 Ch30, Active PLD	MO70 PA00 PC00 Ch30, Active PLD	0.9338
	MO70 PA00 PC00 Ch30, No PLD	MO70 PA00 PC00 Ch30, Active PLD	0.9770
Vesicles	100nm, No PLD	100nm, Active PLD	0.9988
	100nm, No PLD	200nm, No PLD	0.0679
	100nm, No PLD	200nm, Active PLD	0.0679
	100nm, Active PLD	200nm, No PLD	0.0975
	100nm, Active PLD	200nm, Active PLD	0.0975
	200nm, No PLD	200nm, Active PLD	1.0000

Supplementary Table 23: Statistical analysis of PLD activity on LNPs and vesicles via DLS analysis of polydispersity index. Data from Supp. Fig. 14C and E.

Formulation	Group 1	Group 2	p-value
LNP	MO55 PA00 PC15 Ch30, No PLD	MO55 PA00 PC15 Ch30, Active PLD	0.6809
	MO55 PA00 PC15 Ch30, No PLD	MO70 PA00 PC00 Ch30, No PLD	0.1860
	MO55 PA00 PC15 Ch30, No PLD	MO70 PA00 PC00 Ch30, Active PLD	0.7261
	MO55 PA00 PC15 Ch30, Active PLD	MO70 PA00 PC00 Ch30, No PLD	0.8095
	MO55 PA00 PC15 Ch30, Active PLD	MO70 PA00 PC00 Ch30, Active PLD	0.9999
	MO70 PA00 PC00 Ch30, No PLD	MO70 PA00 PC00 Ch30, Active PLD	0.7691
Vesicles	100nm, No PLD	100nm, Active PLD	0.6101
	100nm, No PLD	200nm, No PLD	0.4233
	100nm, No PLD	200nm, Active PLD	0.0076
	100nm, Active PLD	200nm, No PLD	0.9909
	100nm, Active PLD	200nm, Active PLD	0.1996
	200nm, No PLD	200nm, Active PLD	0.3385

References

[1] H. M. G. Barriga, O. Ces, R. V. Law, J. M. Seddon, N. J. Brooks, *Langmuir* **2019**, *35*, 50 16521.

[2] C. Czeslik, R. Winter, G. Rapp, K. Bartels, *Biophysical journal* **1995**, *68*, 4 1423.

[3] J. Y. T. Chong, X. Mulet, L. J. Waddington, B. J. Boyd, C. J. Drummond, *Langmuir* **2012**, *28*, 25 9223.
